# A *Chlamydia trachomatis* CPAF-STING agonist conjugate vaccine administered intramuscularly and intradermally is immunogenic in the pig model

**DOI:** 10.3389/fimmu.2026.1816737

**Published:** 2026-05-01

**Authors:** Leonie Bettin, Christine Unterweger, Maximiliane Dippel, Tamara Borysova, Maria Stadler, Jonathan Harris, James Rozzelle, Daisy Arroyo, Jeff Fairman, Taylor B. Poston, Eric Perouzel, Thierry Lioux, Juan F. Hernandez-Franco, Harm HogenEsch, Andrea Buzanich-Ladinig, Toni Darville, Tobias Käser

**Affiliations:** 1 Department of Biological Sciences and Pathobiology, Centre of Pathobiology, Unit of Immunology, University of Veterinary Medicine Vienna, Vienna, Austria; 2 Clinical Department for Farm Animals and Food System Science, Clinical Centre for Population Medicine in Fish, Pig and Poultry, University of Veterinary Medicine Vienna, Vienna, Austria; 3 Centre for Immunology and Infection Control, Queensland University of Technology, Brisbane, QLD, Australia; 4 Vaxcyte, Inc., San Carlos, CA, United States; 5 Department of Pediatrics, University of North Carolina at Chapel Hill, Chapel Hill, NC, United States; 6 InvivoGen, Toulouse, France; 7 Department of Comparative Pathobiology, College of Veterinary Medicine, Purdue University, West Lafayette, IN, United States; 8 Purdue Institute of Inflammation, Immunology, and Infectious Disease, Purdue University, West Lafayette, IN, United States

**Keywords:** *Chlamydia trachomatis*, conjugate vaccine, CPAF, pig, STING agonist, swine

## Abstract

**Introduction:**

*Chlamydia trachomatis* (*Ct*) is the most common bacterial cause of sexually transmitted infection worldwide. Given the limited effectiveness of current *Ct* screening and treatment programs, alongside the lack of lasting immunity from natural infection, developing an effective vaccine is crucial. Pigs serve as a valuable model for chlamydia research due to their physiological similarity to humans and natural susceptibility to *Chlamydia suis* (*Cs*), a close relative of *Ct*. Thus, we utilized the pig model to evaluate the immunogenicity of three Chlamydial Protease-like Activity Factor (CPAF)-based vaccine candidates. Because stimulator of interferon genes (STING) pathway agonists have shown promise as adjuvants for subunit vaccines but are limited by rapid diffusion from the injection site and poor cellular uptake, we evaluated two improved STING agonist formulations: direct conjugation of the agonist to CPAF (CPAF-STG1151) and adsorption onto phytoglycogen nanoparticles (CPAF/NanoST), and compared their performance to a conventional oil-in-water microemulsion adjuvant (CPAF/IMS1313).

**Methods:**

Pigs received two intramuscular (IM) or two intradermal (ID) doses and adaptive immune responses were assessed weekly by three-color FluoroSpot assays, multi-parameter flow cytometry and anti-CPAF ELISAs.

**Results:**

Among the vaccine candidates tested, the novel CPAF-STG1151 conjugate elicited the strongest systemic T cell response. It induced a robust cell-mediated immune response characterized by IFNγ production with or without TNFα coproduction and this response was equally mediated by all T cell subsets (CD4, CD8 and γδ T cells). The route of administration (IM vs. ID) had no significant effect on either the magnitude or the cytokine profile of the cell-mediated immune response. The highest anti-CPAF IgG serum levels were also observed following vaccination with the CPAF-STG1151 conjugate vaccine candidate.

**Discussion:**

Thus, conjugation of the STING pathway agonist STG1151 to the Chlamydia protein CPAF resulted in a highly immunogenic subunit vaccine. Future studies will determine mucosal administration routes and assess the vaccine efficacy of this promising Ct vaccine candidate.

## 1 Introduction

*Chlamydia trachomatis (Ct****)*** is an obligate intracellular bacterium and the most common sexually transmitted bacterial pathogen worldwide. Most *Ct* infections are asymptomatic, allowing the organism to persist undetected and untreated. Untreated or repeated infections have been linked to serious complications in women, including pelvic inflammatory disease (PID) (1). PID occurs when *Ct* ascends to the upper reproductive tract leading to chronic inflammation and tubal damage that can lead to chronic pain, ectopic pregnancy, and infertility (2). In an effort to reduce clinical *Ct* infections and limit transmission, several vaccines are under development, with three subunit and one mRNA vaccine candidate having progressed to phase I/II clinical trials thus far (3, 4). Nevertheless, no vaccine is available, and continued preclinical evaluation in appropriate animal models is essential to enhance both translatability and the likelihood of clinical success. In contrast to mice, pigs share substantial physiological and anatomical similarities to humans, making them strong biomedical models for human disease and therapeutic developments (5–7). Pigs are particularly relevant for chlamydia research because they are the natural host of *Chlamydia suis* (*Cs*), one of the closest phylogenetic relatives of *Ct* (8). The prevalence of *Cs* in pigs is remarkably high, especially in rectal swabs and fecal samples, pointing toward long-lasting colonization in the large intestine/rectum (9, 10). Although often considered opportunistic, *Cs* has been associated with clinical conditions such as conjunctivitis, pneumonia and reproductive disorders (11, 12). Our previous studies demonstrated that both *Cs* and *Ct* are capable of infecting *Cs*-seropositive pigs and that CD4 T cell responses are cross-reactive (13, 14). This research established *Cs* pre-exposed pigs as a relevant preclinical model for Ct vaccine development, more closely reflecting Phase 3 clinical trial conditions in which populations at increased risk for Ct infection are typically prioritized.

Several studies in mouse models indicate that a protective immune response against *Ct* is dependent on strong T cell responses, in particular, IFNγ secreted by CD4 T cells (Th1) (15–17). Data from *Ct*-exposed women also reveal IFNγ-producing CD4 Th1 cells play a key role in the protection from reinfection (18–20). Although CD4 Th17 cells and their production of IL-17 have been associated with partial protection in mouse models, their overall impact remains uncertain, as they can also contribute to immunopathology observed during chlamydial infections (21–24). Thus, a *Ct* vaccine candidate that elicits a robust Th1-biased immune response is more likely to be effective and safe. However, achieving such a response with a subunit vaccine is challenging and will require one or multiple highly immunogenic proteins as well as safe and effective adjuvants. In a recent study of women infected with *Ct*, T cell responses were evaluated against 33 *Ct* proteins, with Chlamydial Protease-like Activity Factor (CPAF) identified as the most immunoprevalent antigen, recognized by more than 50% of participants (25). CPAF is a serine protease secreted by *Chlamydiaceae* into the host cell cytoplasm, where it assists with processing host proteins that correlate with loss of integrity of the intracellular protective chlamydial vacuole and release of infectious progeny (26). Once released from the host cells, CPAF paralyzes neutrophils to evade the host innate immune response (27).

In our previous study, CPAF was adjuvanted with the STING-activating cyclic dinucleotide 2’3’-c-di-AM(PS)2 (Rp,Rp) (ADU-S100) and administered intramuscularly (IM) and intranasally to pigs (28). Cyclic dinucleotides, like ADU-S100, bind STING leading to the production of proinflammatory cytokines and type I interferons (29). In mice, vaccine formulations containing STING-activating cyclic dinucleotides elicited robust Th1 and Th17 responses (30–32). In particular, a vaccine candidate composed of *Chlamydia muridarum (Cm)* CPAF and ADU-S100, has already been shown to induce memory T cell responses in mice and reduced cervical chlamydial burden upon challenge (33). In pigs, however, the *Ct* CPAF/ADU-S100 admixture vaccine resulted in a rather weak and inconsistent systemic Th1 response and failed to confer protection (28). Although the exact mechanism of this observed species-specific difference is not known, contributing factors could be differences in STING affinity and downstream signaling but also the rapid diffusion and clearance of cyclic dinucleotides from the injection site. This diffusion leaves only a brief window for antigen-presenting cell (APC) activation and limits vaccine potency (34, 35). Moreover, once dispersed, antigen and agonist may no longer be co-delivered to the same cell resulting in suboptimal immunogenicity. Dissemination of STING agonists also contributes to systemic inflammation, which can lead to off-target effects and toxicities (29, 36). Hence, in this current study, we screened improved CPAF-based vaccine formulations that prevent the dissociation of antigen and STING agonist upon administration. We hypothesize that the resulting co-delivery to the same APC promotes a strong Th1-biased immune response.

One promising approach to achieve this co-delivery is the direct conjugation of antigens to immunostimulatory adjuvants. These self-adjuvanted vaccine constructs lead to the uptake of antigen and adjuvant by the same immune cell, which limits diffusion and leads to prolonged and enhanced antigen presentation (37). This approach has been successfully employed with Toll-like-receptor (TLR) ligands to elicit antitumor activity but also in the control of infectious diseases (38). For example, Hanna et al. (39) designed vaccine constructs consisting of full-length ESAT6 protein from *Mycobacterium tuberculosis* fused to the TLR2-ligand Pam_2_CSK_4_ or Pam_3_CSK_4_. After mucosal administration, the vaccine induced strong CD4 T cell responses (Th17) and provided some protection upon aerosol challenge (39). Recently, Wang and colleagues (40) conjugated a STING agonist from the diABZI family to various peptide antigens (OVA, neoantigens and SARS-CoV-2). The constructs were then encapsulated in lipid nanoparticles and evaluated for their potential to augment CD8 T cell responses in mice. They found that the conjugated vaccine elicited markedly stronger CD8 T cell responses than those induced by antigen and STING agonist admixtures or by mixture with conventional adjuvants (40). Another alternative strategy to address the limitations of STING agonists as vaccine adjuvants involves encapsulation or electrostatic loading onto nanoparticles, a method that can extend circulation half-life, improve cellular internalization, and limit rapid diffusion (29, 41). In fact, the corn-based cationic α-D-glucan nanoparticle (Nano-11) has been successfully combined with STING agonists c-di-AMP and ADU-S100. The resulting vaccine formulations significantly enhanced immune responses against the model antigen OVA and inactivated IAV, particularly when administered intradermally (ID) (42–44). Intradermal administration offers immunological advantages due to the high density of APCs in the dermis, with the added potential for dose sparing and needle-free administration (45, 46).

Hence, in this study, the full-length inactivated protein CPAF from *Ct* was either conjugated to a novel STING agonist STG1151 or adsorbed onto the nanoparticle-based combination adjuvant NanoST (Nano11 + ADU-S100) and administered through IM or ID routes. STG1151 is an innovative protein-conjugatable STING ligand that has been designed by InvivoGen with a cleavable linker and a spacer that minimizes steric hindrance. Upon cellular uptake of CPAF-STG1151, the linker is cleaved by Cathepsin B allowing release of the free STING cyclic dinucleotide agonist solely in the intracellular space. Both vaccine candidates, CPAF-STG1151 and CPAF/NanoST, were compared to the commercially available oil-in-water microemulsion adjuvant Montanide™ IMS 1313 N VG PR (IMS 1313).

## 2 Materials and methods

### 2.1 Animal trial

**Figure 1:**
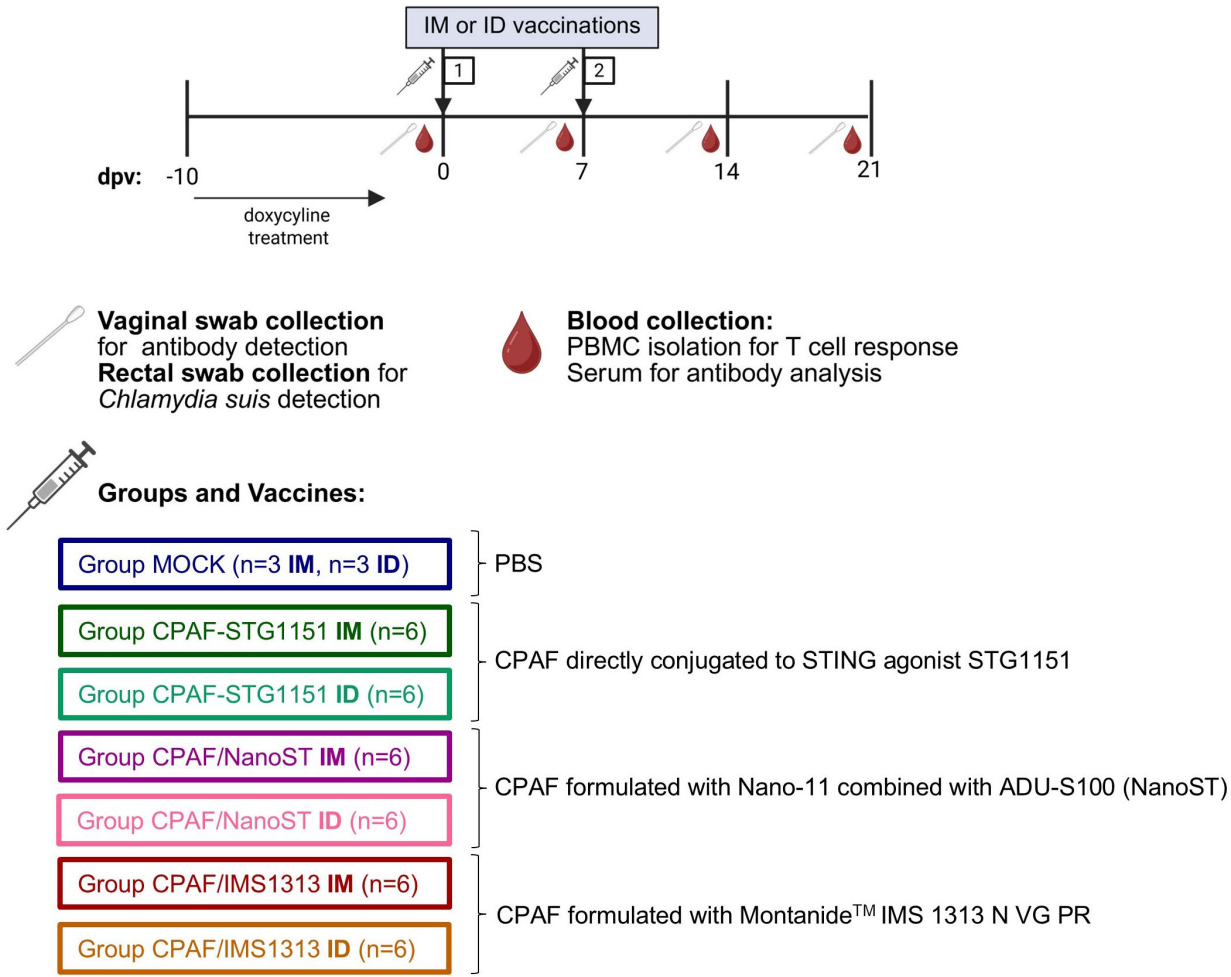
*Ct* CPAF vaccination trial layout. Upon arrival, the *Chlamydia suis* (*Cs)* pre-exposed outbred pigs were treated with doxycycline for 8 days to eliminate ongoing *Cs* colonization. Forty-two 4-week-old pigs were allocated to seven groups as outlined above and vaccinated on day 0 and 7 by either intramuscular (IM) or intradermal (ID) administration using a needle-free injection system (PharmaJet Tropis^®^ ID). Each group consisted of four females and two males. Throughout the trial, blood and swabs (vaginal and rectal) were collected weekly. Vaginal swabs were placed in PBS for the downstream detection of antibodies, while rectal swabs were placed in sucrose phosphate buffer to confirm the *Cs* negative status throughout the trial. dpv= days post (first) vaccination. Figure created in BioRender. Kaeser, T. (2026) https://BioRender.com/ekw2p5k.

The animal trial was carried out as depicted in **Figure 1**. Forty-two 4-week-old *Cs* pre-exposed outbred pigs from the VetFarm (Austria) were brought to the University of Veterinary Medicine Vienna. Rectal swabs were collected at arrival, and a *Chlamydiaceae* qPCR, followed by a *Cs*-specific qPCR assay was performed (47), confirming that all pigs showed rectal shedding of *Cs*. Piglets were randomly distributed into groups while accounting for litter, body weight, and sex to ensure balanced distribution across groups and to minimize litter and weight biases. This resulted in the seven experimental groups shown in **Figure 1** with 4 females and 2 males per group (n = 6 per group). Pigs were administered 12.5 mg/kg body weight of doxycycline (Pulmodox 5%, Virbac, France) daily orally for eight days to eliminate ongoing *Cs* colonization. A *Cs*-specific qPCR performed on rectal swabs confirmed that antibiotic-treated pigs were negative for *Cs*. Rectal swab sampling followed by qPCR was repeated at 7-day intervals throughout the study to monitor *Cs* colonization status. All pigs remained negative for *Cs* for the duration of the trial (data not shown). Three days after the end of the antibiotic treatment, pigs received the first intramuscular (IM) or intradermal (ID) vaccination followed by a second IM or ID vaccination seven days later. Pigs vaccinated IM at the first dose were also vaccinated intramuscularly at the second dose, and the same vaccination route was maintained for ID groups. IM vaccinations were given with needle and syringe, while ID vaccines were administered using the PharmaJet Tropis needle-free injection system (PharmaJet, Golden, CO, USA). IM injections were given in the lateral cervical musculature, specifically in the region of the trapezius and brachiocephalicus muscles, two fingers caudal to the base of the ear. ID injections were given in the same region. No adverse reactions were observed, independent of the vaccine formulation or the route of administration.

Blood (all animals) and vaginal swabs (females only) were collected weekly (days 0, 7, 14, and 21 post first vaccination). Blood samples were used to obtain sera and peripheral blood mononuclear cells (PBMCs) for analyzing the systemic humoral and cellular anti-CPAF immune responses, respectively. Swabs were used to analyze the local anti-CPAF antibody response. This animal trial was approved by the Ethics and Animal Welfare Committee of the University of Veterinary Medicine, Vienna, in accordance with the University’s guidelines for Good Scientific Practice and authorized by the Austrian Federal Ministry of Education, Science and Research (BMBWF 2023-0.588.126) in accordance with current legislation.

### 2.2 Vaccine antigen production and formulation

Pigs were assigned to receive one of the vaccine formulations described below. Animals in the MOCK group were administered PBS. The final dose volume was 1 mL per pig for IM vaccination and 100 µL per pig for ID vaccination.

#### 2.2.1 CPAF-STG1151 conjugate vaccine

To express the Chlamydia (Chlamydia trachomatis) Protease Activity Factor (CPAF Ct) variant used for conjugation, a codon-optimized synthetic gene (ATUM, Inc.) was cloned in a pUG-based plasmid behind a T7 promoter. The CPAF Ct variant has a TEV-cleavable N-terminal Histidine tag and five point mutations (1): the catalytic Serine was changed to Alanine (S499A) to abolish proteolytic acitivity, (2&3) K232Q and R235Q remove clip sites due to exogenous proteolytic activity (4) C584A reduces disulfide-mediated aggregation and (5) a para-azido phenylalanine (pAMF) non-native amino acid (Asymchem, Boston) was introduced at F129 (F129pAMF) for covalent conjugation of DBCO-derivatized adjuvant. The F129pAMF CPAF Ct variant was expressed in a cell free, *in-vitro* translation system (XpressCF^®^, Sutro Biopharma; international patent application WO2015054587A1). The XpressCF^®^ cell-free protein synthesis system can be obtained through partnership with Sutro Biopharma. CPAF Ct (F129pAMF) was purified by Ni affinity chromatography (Cytiva). The purified protein was conjugated to a DBCO-derivatized STING agonist (STG1151, InvivoGen) using Cu-free Click chemistry; CPAF Ct F129pAMF variant was mixed with 1.5 equivalents of STG1151 and then incubated at room temperature with gentle agitation. The reaction was monitored by competition with DBCO-Tamra fluorescent dye (Lumiprobe Corp.). The disappearance of fluorescent signal indicated the reaction went to completion in two hours. Unreacted STG1151 was removed with a desalting column. On the day of vaccination, 30 µg of CPAF-STG1151 per dose was diluted in PBS to the final volumes outlined above. For both IM and ID administration, the dosages of CPAF-STG1151 were identical. STG1151 is a conjugatable STING agonist derived from the cyclic dinucleotide CL845 (cAIM(PS) Difluor (Rp)), an analog of the STING agonist CL656 (cAIM(PS)_2_ Difluor (Rp/Sp)) (InvivoGen).

#### 2.2.2 CPAF/NanoST vaccine

Nano-11 was produced using a previously described protocol (48). In brief, phytoglycogen (PG) nanoparticles from sweet corn were reacted with octenyl succinic anhydride (OS) and (3-chloro-2-hydroxypropyl)-trimethylammonium chloride (CHPTAC), yielding PG-OS-CHPTAC (Nano-11). The resulting positively charged Nano-11 particles facilitate electrostatic adsorption of negatively charged protein antigens, forming stable antigen – Nano-11 complexes.

The recombinant inactivated CPAF vaccine antigen was produced in *E. coli* strain BL21 DE3 as described previously (45). In short, the CPAF sequence was derived from WP_015506580, using a codon-optimized open reading frame that excluded the first 26 residues to improve expression, while proteolytic activity was disrupted by S499A substitution. This modified sequence was cloned into the pRSETA vector. Following expression, the protein was purified with Cobalt agarose (Talon ^®^), and residual LPS was eliminated through cloud-point detergent extraction. Subsequently, the purified CPAF protein was lyophilized for storage. The STING agonist ADU-S100 (2’3’-c-di-AM(PS)2 (Rp,RP)) was prepared according to the manufacturer’s instructions (InvivoGen, San Diego, CA).

The final vaccine was prepared the morning of vaccination by combining 1 mg Nano-11 with 50 μg ADU-S100 per dose for 1 h at room temperature to create the combination adjuvant NanoST. The adsorption of ADU-S100 onto Nano-11 has been previously established and validated by ultraperformance liquid chromatography/tandem mass spectrometry indicating over 80% adsorption of ADU-S100 onto Nano-11 (42, 43). The recombinant CPAF (30 μg per dose) was subsequently adsorbed onto NanoST for an additional hour. Recombinant CPAF, expressed as described above, has a theoretical isoelectric point (pI) of 5.4, which means that at physiological pH (7.4) it carries a net negative charge, facilitating its adsorption onto the positively charged NanoST particles. For both IM and ID administration, the dosages of NanoST and CPAF were identical.

#### 2.2.3 CPAF/Montanide™ IMS1313 N VG PR vaccine

The recombinant CPAF was produced as described above and then combined with the Montanide™ IMS 1313 N VG PR adjuvant shortly before administration according to manufacturer’s instructions (Seppic, Courbevoie, France). The adjuvant was used at a ratio of 1:1. Each pig received 30 μg of CPAF per dose (IM and ID).

### 2.3 Cell isolation, swabs and sera

PBMCs were isolated from heparinized blood by density gradient centrifugation using lymphocyte separation medium (Pancoll, PAN Biotech, Aidenbach, Germany) and SepMate™ tubes (StemCell, Vancouver, BC, Canada) according to manufacturer’s instructions. A red blood cell (RBC) lysis step was included using RBC lysis solution (Thermo Fisher Scientific, Waltham, MA). After isolation, a Sysmex XP 300 hematology analyzer (Sysmex Europe GmbH, Norderstedt, Germany) was used to count cells and fresh PBMCs were plated for *in vitro* restimulation to study the anti-CPAF T cell response. Remaining PBMCs were cryopreserved in freezing media (50% RPMI 1640, 40% FBS, 10% DMSO) and stored in -150 °C. Serum tubes were rested for >30 min, then spun at 2,000 x g for 10 min, after which serum was aliquoted and frozen at -20 °C for downstream anti-CPAF IgG analysis. For the vaginal swab collection, the vulva was cleaned, and the swab was rotated on the vaginal epithelium. The vaginal swab was placed in 1ml of PBS and mixed by vortexing before the swab was removed. The samples were then frozen at -20 °C for future antibody analysis. Rectal swabs were collected by inserting swabs into the pig’s rectum. These swabs were then placed in 1ml of sucrose phosphate (SP) buffer and stored -80 °C. Swabs placed in SP buffer were used to monitor chlamydial burden throughout the trial.

### 2.4 IFNγ/TNFα/IL-17A FluoroSpot

Triple-color FluoroSpots were performed according to the manufacturer’s instructions (MabTech, Nacka Strand, Sweden). In brief, plates with low-fluorescent PVDF membranes were activated with 35% ethanol, followed by five washes with sterile water and coated overnight at 4 °C with anti-IFNγ (pIFNγ-I), anti-TNFα (MT32E9) and anti-IL-17A (MT49A7) capture antibodies. The following day, plates were blocked for at least 1h with cell culture medium (RPMI 1640 supplemented with 10% FCS and Penicillin-Streptomycin). The blocking medium was removed and fresh medium with or without stimuli was added. Concanavalin A (ConA, 3 µg/mL) served as the positive control, while a CPAF peptide pool (1 µg/mL per peptide) was used to assess antigen-specific T cell responses. The CPAF pool consisted of 148 15-mer peptides with an 11-aa overlap and was prepared according to the manufacturer’s instructions (PepMix™ CPAF, JPT Peptide Technologies, Berlin, Germany). Following the blocking and adding of stimuli to the plate, freshly isolated PBMCs were seeded at 2.5 x 10^5^ cells per well. Each sample was analyzed in triplicate and incubated for 40 h at 37 °C and 5% CO2. After incubation, cells were washed away, and 100 µL of anti-IFNγ BAM (P2C11), anti-TNFα WASP (MT859), and anti-IL-17A biotin (MTP853) diluted in PBS containing 0.1% bovine serum albumin (BSA) was added to each well. Plates were incubated for 2 h at room temperature and washed five times with PBS before detection reagents (anti-BAM-490, anti-WASP-640, and SA-550) were added for an additional 1 h incubation at room temp. Plates were washed as described above, followed by the addition of 50 µL fluorescence enhancer per well for 10 min. After discarding the enhancer, plates were dried in the dark and analyzed using an ELISpot/FluoroSpot reader system (IRIS2, MabTech). The IRIS reader software measures not only spot-forming units (SFU), but also the spot size and calculates the relative spot volume. The analysis of average relative spot volumes (IFNγ) was performed on responder samples from 14 and 21 dpv. A responder threshold was defined as the mean of the media control wells plus two standard deviations (SDs): 3.232 SFU/500k (mean of media control wells) + 2 x 3.149 SFU/500k (SD of media control wells) = 9.5 SFU/500k.

### 2.5 *In vitro* stimulation and flow cytometry staining

**Figure 2:**
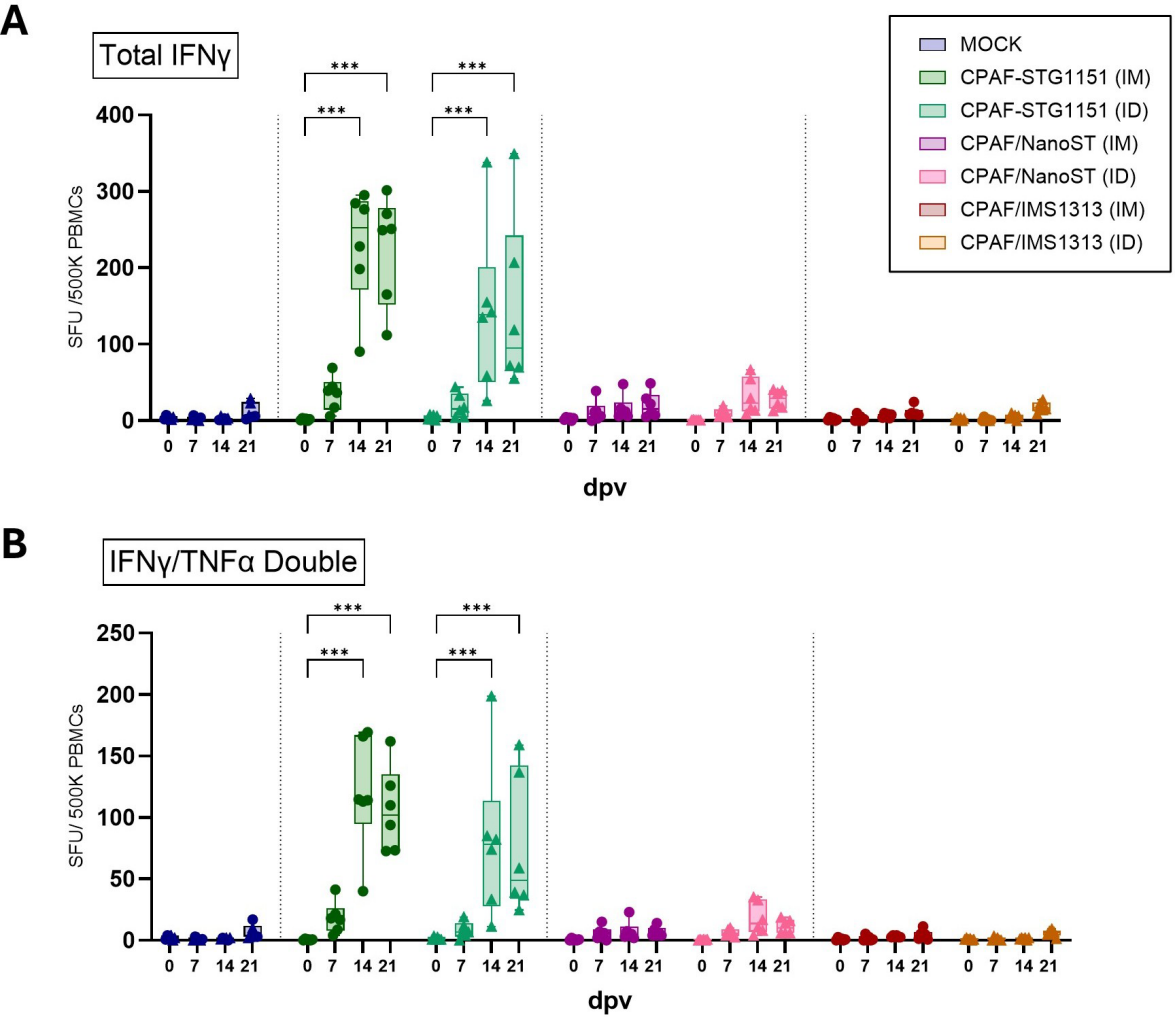
After CPAF restimulation, PBMCs from CPAF-STG1151 vaccinated pigs exhibited the highest frequencies of IFNγ and IFNγ/TNFα-producing cells. **(A)** Total IFNγ production by PBMCs was measured by FluoroSpot assay after *in vitro* CPAF restimulation of freshly isolated PBMCs. **(B)** shows the spot count for IFNγ/TNFα double positive cells. Each symbol represents an individual animal (n=6 per group). In the MOCK group, three pigs received intramuscular (IM) PBS injections (circle), and three pigs received intradermal (ID) PBS injections (triangle). Statistical analysis was performed via GraphPad using 2-way ANOVA and Tukey multiple comparisons test. Statistical analysis of within-group comparisons are shown. ***p < 0.001. dpv = days post (first) vaccination. SFU = Spot forming units.

For intracellular cytokine staining of IFNγ, TNFα, and IL-17A, round-bottom 96-well plates were seeded in quadruplicate with 5 × 10^5^ freshly isolated PBMCs per well in culture medium (RPMI 1640; PAN Biotech) supplemented with 10% fetal bovine serum (FBS; Merck KGaA, Darmstadt, Germany) and Penicillin–Streptomycin (PAN Biotech). PBMCs were either left unstimulated (negative control) or stimulated overnight with the CPAF peptide pool described above (1 µg/mL per peptide; JPT Peptide Technologies). Cells stimulated with ConA (3 µg/mL) served as positive controls. After 14 h of culture, Brefeldin A (BD GolgiPlug™, BD Biosciences, San Jose, CA, USA) was added at a final concentration of 1 μg/mL for 4h. Quadruplicates were then pooled and stained for flow cytometry as described below. Stimulated PBMCs were first surface stained with primary monoclonal antibodies, including directly conjugated antibodies as listed in **Table 1**. This was followed by staining with secondary antibodies and the fixable Viability Dye eFluor™ 780 (Thermo Fisher) to exclude dead cells. Intracellular staining was then performed using the BD Cytofix/Cytoperm™ Fixation/Permeabilization Kit (BD Biosciences, San Jose, CA, USA) according to the manufacturer’s instructions. All staining steps were carried out at 4 °C for 20 min (surface and secondary staining) or 30 min (intracellular staining). Antibodies were titrated prior to use, and compensation was calculated from single-color controls. Technical details of all antibodies are provided in **Table 1**. A minimum of 0.9 × 10^6^ events per sample were acquired on a Beckman Coulter CytoFLEX LX™ (U3-V5-B3-Y5-R3-I2 laser configuration). Data were analyzed in FlowJo™ Software (v10.8.1; BD Biosciences) using gates defined by fluorescence minus one (FMO) controls. Dead cells and doublets were excluded as shown in **Supplementary Figure 1**; **Figure 2**.

**Table 1:** Primary antibodies and secondary reagents used for flow cytometric analysis.

Antigen	Clone	Isotype	Fluoro- chrome	Labeling strategy	Primary antibody source	Secondary antibody source
CPAF specific T-cell response: Cytokine production						
CD3	PPT3	mIgG1	FITC	Directly conjugated	Southern Biotech	-
CD4	74-12-4	mIgG2b	BV421	Secondary antibody	In house	Jackson ImmunoResearch
CD8α	76-2-11	mIgG2a	BUV395	Biotin-Streptavidin	Thermo Fisher	Biolegend
CCR7	3D12	rIgG2a	BB700	Directly conjugated	BD Bioscience	–
TCRγδ	PGBL22A	mIgG1	AF647*	Directly conjugated	Kingfisher	Thermo Fisher
Live/Dead	–	–	eFlour780	–	Invitrogen	–
TNFα	Mab11	mIgG2a	BV605	Directly conjugated	Biolegend	–
IFNγ	P2G10	mIgG1	PE	Directly conjugated	BD Biosciences	–
IL-17A	ebio64DEC17	mIgG1	PE-Cy7	Directly conjugated	ThermoFisher	–

*Directly labeled with Alexa Fluor™ 647 Antibody Labeling Kit (Thermo Fisher).

### 2.6 Anti-CPAF IgG/IgA ELISA

CPAF IgG ELISAs were performed as detailed in Bettin et al. (28). In brief, Nunc™ MaxiSorp™ flat-bottom 96-well plates (Thermo Fisher Scientific) were coated overnight at 4 °C with 10 µg/mL of CPAF protein in a carbonate coating buffer, using the same CPAF preparation that was used in the respective vaccine. The next morning, plates were washed and then blocked for at least 1 h at room temperature (RT) using 1% BSA in PBS. Sera from pigs were diluted 1: 1,000 in assay buffer (PBS + 0.01% Tween-20 + 0.1% BSA) or prepared as a twofold dilution row from 1:250 to 1: 8,000, while vaginal swab eluates were used undiluted. Samples were added and the plate was incubated overnight at 4 °C. The following morning, the horseradish peroxidase-conjugated anti-pig IgG (1:200,000) or anti-pig IgA (1:100,000) detection antibodies were added to the wells for 2 h at RT (Bethyl Laboratories Inc., Montgomery, TX; A100-117P). This was followed by the addition of substrate (3,3′,5,5′-Tetramethylbenzidine) for 30min at RT. The reaction was stopped by the addition of stop solution (sulphuric acid). The color reaction was quantified by optical density (OD) measurements at 450/620 nm using a Tecan Sunrise ELISA reader (Tecan, Männedorf, Switzerland). All assays were run in duplicates, and each plate included a positive and negative control sample. To minimize plate-to-plate variability, all samples within a given experimental group (all timepoints from the same animals) were analyzed on the same ELISA plate. Additionally, using the positive control serum, inter-assay variability was assessed and yielding an inter-assay coefficient of variation (%CV) of <15% [10.21% for the coating with the CPAF preparation used in the STG1151 conjugate vaccine and 13.58% for the coating with the CPAF preparation used in the NanoST and IMS1313 vaccine].

### 2.7 Statistical analysis

Statistical analyses were performed using GraphPad Prism version 10.6.1. Prior to applying parametric tests, data were assessed for normality using the Shapiro-Wilk test, with distributions considered normal when p ≥ 0.05. Depending on the experiment, either one-way or two-way ANOVA was applied, as specified in the figure legends. When significant differences were detected, pairwise comparisons were conducted using Tukey’s multiple comparison test. Statistical significance was defined as p ≤ 0.05 (∗), p ≤ 0.01 (∗∗), and p ≤ 0.001 (∗∗∗).

## 3 Results

### 3.1 Vaccination with CPAF-STG1151 conjugate induces a strong systemic IFNγ response

Following group allocation and vaccination as outlined in **Figure 1**, weekly blood samples were collected. Antigen-specific cytokine production by PBMCs was then assessed using FluoroSpot assays, which allowed for the simultaneous detection of IFNγ, TNFα, and IL-17A. Cytokine secretion was analyzed in unstimulated wells (media control) and after 40h stimulation with CPAF peptide pool; ConA served as a positive control. Representative images of the wells are shown in **Supplementary Figure 1A**. Spot-forming units (SFU) were counted and classified as single-, double-, or triple-positive based on spatial colocalization of cytokine signals (**Supplementary Figure 1B**). While PBMCs from MOCK-vaccinated pigs produced IFNγ only at background levels (mean 5 SFUs), PBMCs from CPAF-STG1151 vaccinated pigs released IFNγ in response to CPAF stimulation from 14 days post (first) vaccination (dpv) onwards regardless of administration route (**Figure 2A**). Although not statistically significant, IM administration of the CPAF-STG1151 vaccine appeared to induce a slightly higher and more consistent IFNγ response compared to ID administration (mean of 229 and 142 SFUs respectively at 14 dpv). The response observed following ID administration of CPAF-STG1151 appears to be largely driven by one to two responder animals. The other vaccine candidates tested, CPAF/NanoST and CPAF/IMS1313, elicited significantly lower IFNγ responses compared to CPAF-STG1151 (administered IM or ID) at 14 and 21 dpv. Both the CPAF/NanoST and the CPAF/IMS1313 groups were not statistically different from the MOCK-vaccinated group on any of the timepoints included in the study. Despite the low-level response, CPAF/NanoST did show a trend toward increased IFNγ production 14 dpv when administered intradermally, while the oil-in-water emulsion CPAF/IMS1313 showed no IFNγ response over background levels at any timepoint. The analysis of double-positive cells (IFNγ and TNFα) revealed a similar pattern for all vaccine candidates (**Figure 2B**). The CPAF-STG1151 conjugate vaccine induced the highest count of IFNγ^+^TNFα^+^ spots 14 and 21 dpv. Among the cytokines analyzed, TNFα was produced by the highest number of cells. However, elevated background levels were noted in media control wells and varied across sampling days. In fact, when PBMCs from MOCK-vaccinated pigs produced cytokines in response to CPAF stimulation, they were almost exclusively TNFα single-secreting cells suggesting that these TNFα single-positive cells likely reflect bystander or non-specific activation rather than CPAF-specific responses (**Supplementary Figures 2A, B**). Nevertheless, after background correction, an increase in total TNFα production by PBMCs can be observed for pigs vaccinated with the CPAF-STG1151 conjugate (IM, ID) or with the CPAF/NanoST vaccine (ID) (**Supplementary Figure 3A**).

Overall, vaccination shifted the cytokine profile from predominantly TNFα single-secreting cells toward an increased contribution of IFNγ single-secreting and IFNγ/TNFα double-secreting cells as shown in **Supplementary Figure 2B**. The greatest shift was observed for the CPAF-STG1151 conjugate vaccine (IM), followed by the CPAF-STG1151 vaccine administered ID. The CPAF/NanoST vaccination still resulted in some IFNγ contribution to the cytokine profile, whereas CPAF/IMS1313 closely resembled the profile of MOCK-vaccinated pigs, with a strong bias toward TNFα single-secreting cells (**Supplementary Figure 2B**). Other cytokine combinations, especially those including IL-17A were not observed regardless of the vaccine administered (**Supplementary Figure 2B**). This was confirmed by analyzing the IL-17A spot count over time and for each group. The number of SFUs/500K PBMCs was low (< 35 SFUs) throughout, and no vaccine-related pattern was observed (**Supplementary Figure 3B**). Together, these findings indicate that, in particular, the CPAF-STG1151 vaccine induced CPAF-specific PBMC cytokine responses characterized by IFNγ production with or without co-production of TNFα.

### 3.2 Vaccine- and time-dependent quantitative differences in IFNγ secretion

**Figure 3:**
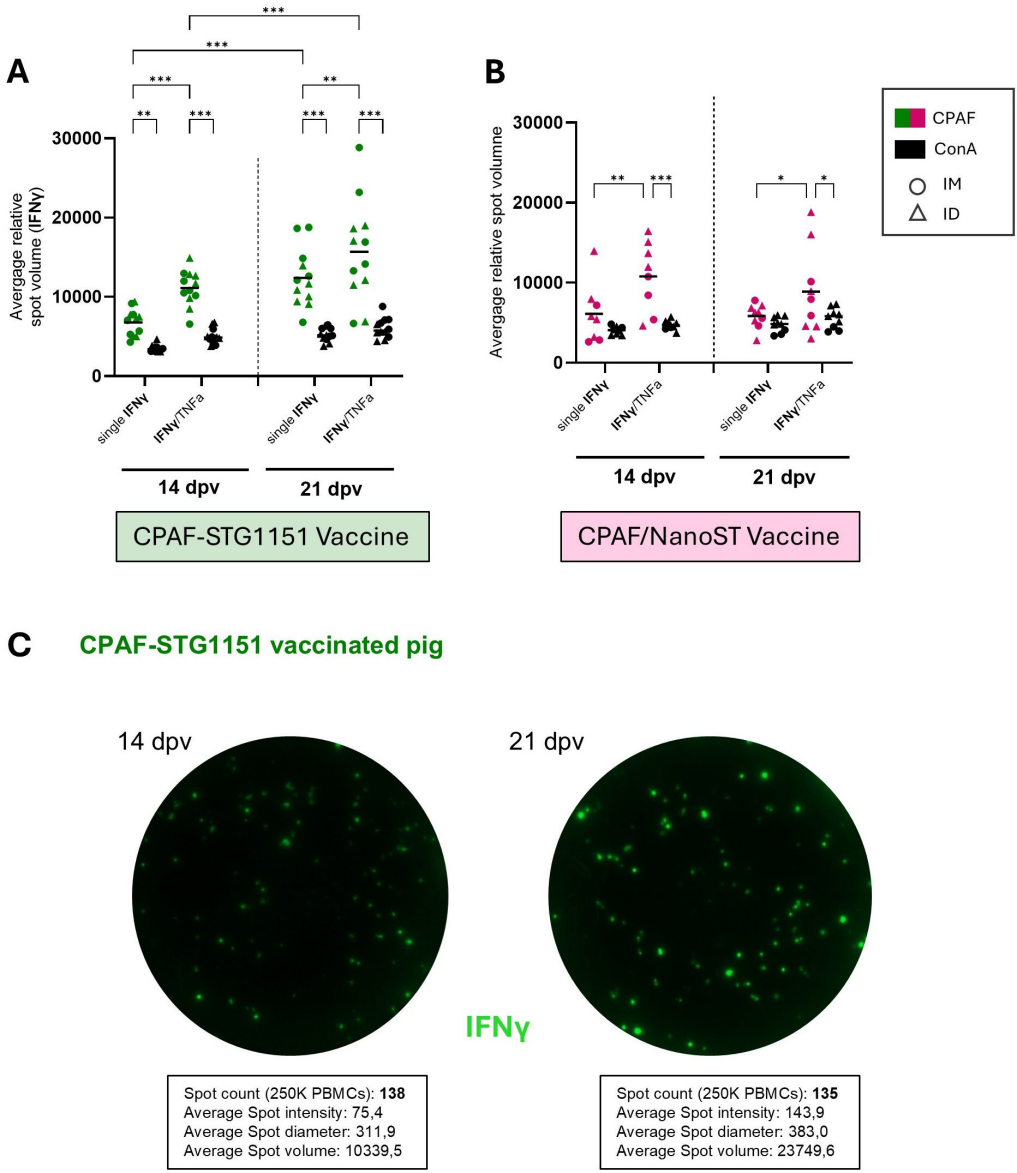
Average IFNγ spot volume increases for CPAF-STG1151 vaccinated animals between 14 and 21 dpv. The graphs show the average IFNγ spot volume in response to CPAF peptide pool stimulation and the positive control (ConA) at 14 and 21 dpv for pigs vaccinated with CPAF-STG1151 **(A)** or CPAF/NanoST **(B)**. IM and ID vaccinated pigs were combined for this analysis (n= 12 CPAF-STG1151 vaccine; n= 8 CPAF/NanoST vaccine at 14dpv and n= 9 at 21 dpv). Only responder samples were included in this analysis. The responder cut-off was set as the mean of the media control wells plus two standard deviations = 9.5 SFU/500k PBMCs. Representative FluoroSpot wells are shown in **(C)**. IFNγ positive spots (green) are shown for one CPAF-STG1151 vaccinated animal 14 and 21 dpv upon CPAF peptide pool stimulation. Statistical analysis was performed via GraphPad using 2-way ANOVA and Tukey multiple comparisons test. *p < 0.05, **p < 0.01, ***p < 0.001. dpv = days post (first) vaccination.

In addition to enumerating IFNγ-producing cells, we analyzed the average relative spot volume (RSV), which reflects the relative amount of secreted IFNγ per cell (**Figure 3**). Specifically, the RSV of IFNγ was assessed for both single IFNγ spots and IFNγ/TNFα double-positive spots. This analysis focused on the two vaccine candidates and timepoints that elicited the highest IFNγ responses (CPAF-STG1151 and CPAF/NanoST at 14 and 21 dpv). Responder samples were defined as those with SFU counts exceeding the mean in media control wells plus two standard deviations (> 9.5 SFU/500k PBMCs).

Using this threshold, the responder rate in the CPAF-STG1151 groups was 100% at both 14 and 21 dpv. In comparison, the CPAF/NanoST groups showed lower responder rates, with 66% at 14 dpv and 75% at 21 dpv.

While stimulation with the mitogen ConA induced a high number of IFNγ positive spots (**Supplementary Figure 4**), the average RSV values were lower than those observed with CPAF stimulation (**Figures 3A, B**). Across both vaccines, cells secreting only IFNγ exhibited lower IFNγ spot volumes than cells secreting both IFNγ and TNFα, suggesting that double-secreting cells released higher amounts of IFNγ (**Figures 3A, B**). Interestingly, while the RSV of ConA-stimulated cells remained unchanged over time, animals vaccinated with the CPAF-STG1151 conjugate showed increased IFNγ spot volumes between 14 and 21 dpv - for both single IFNγ and double IFNγ/TNFα producing cells (**Figure 3A**). In contrast, responding CPAF/NanoST vaccinated pigs exhibited stable or slightly decreased spot volumes between 14 and 21 dpv (**Figure 3B**). The higher spot volume observed for CPAF-STG1151 vaccinated pigs at 21 dpv also corresponds to greater spot intensity and diameter as shown for a representative pig at 14 and 21 dpv (**Figure 3C**). Although the number of IFNγ positive spots per well remained largely unchanged (138 and 135, respectively), the average spot intensity, diameter and volume increased noticeably between day 14 and day 21 (**Figure 3C**). The combined analysis of spot numbers and volumes suggests that in CPAF-STG1151 vaccinated animals, the number of cells responding to CPAF remains stable between day 14 and 21 post vaccination; yet the amount of IFNγ produced per responding cell increases over this period.

### 3.3 CD4, CD8, and γδ T cell subsets are involved in the antigen-specific cytokine response

**Figure 4:**
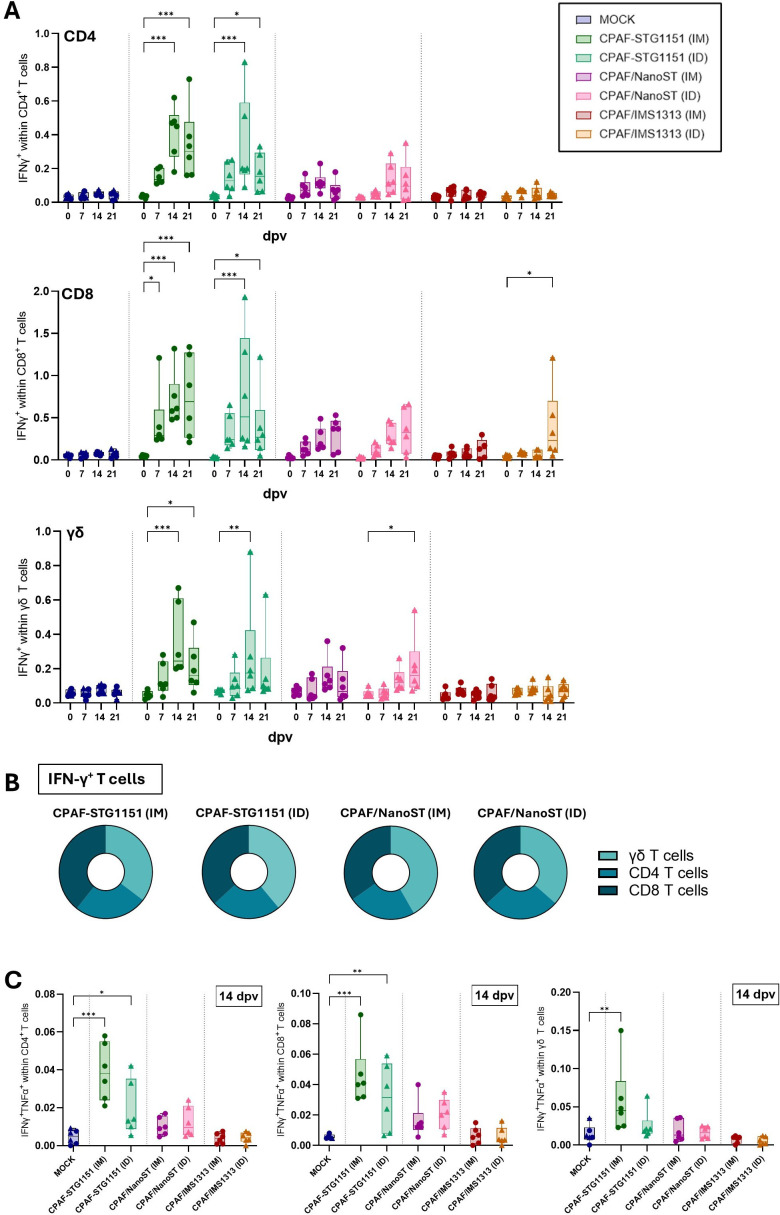
Antigen-specific IFNγ production by T cell subsets. Freshly isolated PBMCs were restimulated overnight with CPAF peptide pool. During data analysis, dead cells and doublets were excluded, as shown in **Supplementary Figure 5A**. After identification of T cell subsets, their IFN-γ production was analyzed. Panel **(A)** shows the percentage of IFN-γ positive cells within CD4, CD8 or γδ T cells at different time points (0,7,14 and 21 dpv). **(B)** Donut plots illustrate the contribution of each T cell subset (CD4, CD8, γδ T cells) to the total IFNγ^+^ T cells at 14 dpv. Data are shown as the mean calculated from n = 6 animals per group. The corresponding gating strategy used to define subsets is provided in **Supplementary Figure 6**. **(C)** PBMCs from 14 dpv were analyzed for IFNγ and TNFα co-production within T cell subsets (CD4, CD8 and γδ T cells). Representative flow cytometry plots are shown in **Supplementary Figure 5B**. Each symbol represents data from one individual pig (n=6 per group). In the MOCK group, three pigs received intramuscular (IM) PBS injections (circle), and three pigs received intradermal (ID) PBS injections (triangle). Statistical analysis in **(A, C)** was performed via GraphPad using 2-way ANOVA and Tukey multiple comparisons test. Only the statistical analysis of within-group comparisons are shown in **(A)**. *p < 0.05, **p < 0.01, ***p < 0.001. dpv = days post (first) vaccination.

While FluoroSpot assays (**Figures 2**, **3**) revealed a vaccine-induced cytokine response within total PBMCs, we next used flow cytometry to specifically analyze the CPAF-specific T cell responses. Freshly isolated PBMCs were restimulated *in vitro* with a CPAF peptide pool and stained for flow cytometry analysis using antibodies and fluorochromes listed in **Table 1**. The gating strategies are shown in **Supplementary Figures 5**, **6**. Consistent with the FluoroSpot assay results, MOCK-vaccinated pigs showed no increase in IFNγ production by CD4, CD8 or γδ T cells over time and the strongest T cell response was observed for CPAF-STG1151 vaccinated animals (**Figure 4A**). Although the administration route (IM vs ID) did not significantly impact the T cell response, IM administration of the CPAF-STG1151 vaccine showed a slight tendency toward stronger and more consistent IFNγ production by T cells compared to ID delivery, further supporting the FluoroSpot results (**Figure 4A**). Although less pronounced than with the CPAF-STG1151 vaccine, CPAF/NanoST vaccination (IM and ID) showed a trend toward increased IFNγ production by CD4, CD8 and γδ T cells at 14 and 21 dpv (**Figure 4A**). The use of the oil-in-water emulsion adjuvant (CPAF/IMS1313) did not elicit antigen-specific IFNγ production by T cells above background levels. Overall, if an antigen-specific response in terms of IFNγ production was present, it was seen within all T cell subsets (CD4, CD8, γδ T cells). To determine which T cell subset contributes most to the vaccine-induced T cell response, we first gated on IFNγ positive T cells and then analyzed the relative contributions of CD4, CD8, and γδ T cells (**Supplementary Figure 6**). For the vaccine candidates that elicited a T cell response (CPAF-STG1151 and CPAF/NanoST), this analysis revealed that all T cell subsets contributed equally to the total IFNγ produced by T cells at 14 dpv regardless of administration route (**Figure 4B**).

TNFα production by T cells showed higher background levels and greater variability between sampling days compared to IFNγ production (**Supplementary Figure 7**). Nonetheless, CD4 T cells from CPAF-STG1151 and CPAF/NanoST vaccinated animals produced TNFα in response to CPAF stimulation at 14 dpv, exceeding background levels (**Supplementary Figure 7A**). Animals receiving the CPAF-STG1151 vaccine (IM and ID) also exhibited elevated TNFα production by CD4 T cells on day 21. No clear trend in TNFα production was observed in CD8 or γδ T cells (**Supplementary Figures 7B, C**). The staining panel also included IL-17A; however, no IL-17A production was detected within T cells following restimulation with CPAF (data not shown). In addition to evaluating total IFNγ and TNFα production within T cells, we assessed multifunctional T cells that simultaneously produce both IFNγ and TNFα (**Figure 4C**). At 14 dpv, IM vaccination and, to a lesser extent, ID vaccination with CPAF-STG1151 led to an increased frequency of IFNγ^+^TNFα^+^ cells within CD4, CD8 and γδ T cells compared to MOCK-vaccinated pigs (**Figure 4C**). In contrast, neither CPAF/NanoST nor CPAF/IMS1313 induced significant multifunctional T cell responses compared to MOCK-vaccinated animals, regardless of the administration route (**Figure 4C**). Overall, these data show that CPAF-STG1151 vaccination consistently elicited strong CPAF-specific IFNγ responses within CD4, CD8 and γδ T cells, whereas the other formulations induced only minimal or no T cell responses.

### 3.4 IFNγ^+^ CD4 T cells shift toward a T_EM_ phenotype in CPAF-STG1151 and CPAF/NanoST vaccinated animals

**Figure 5:**
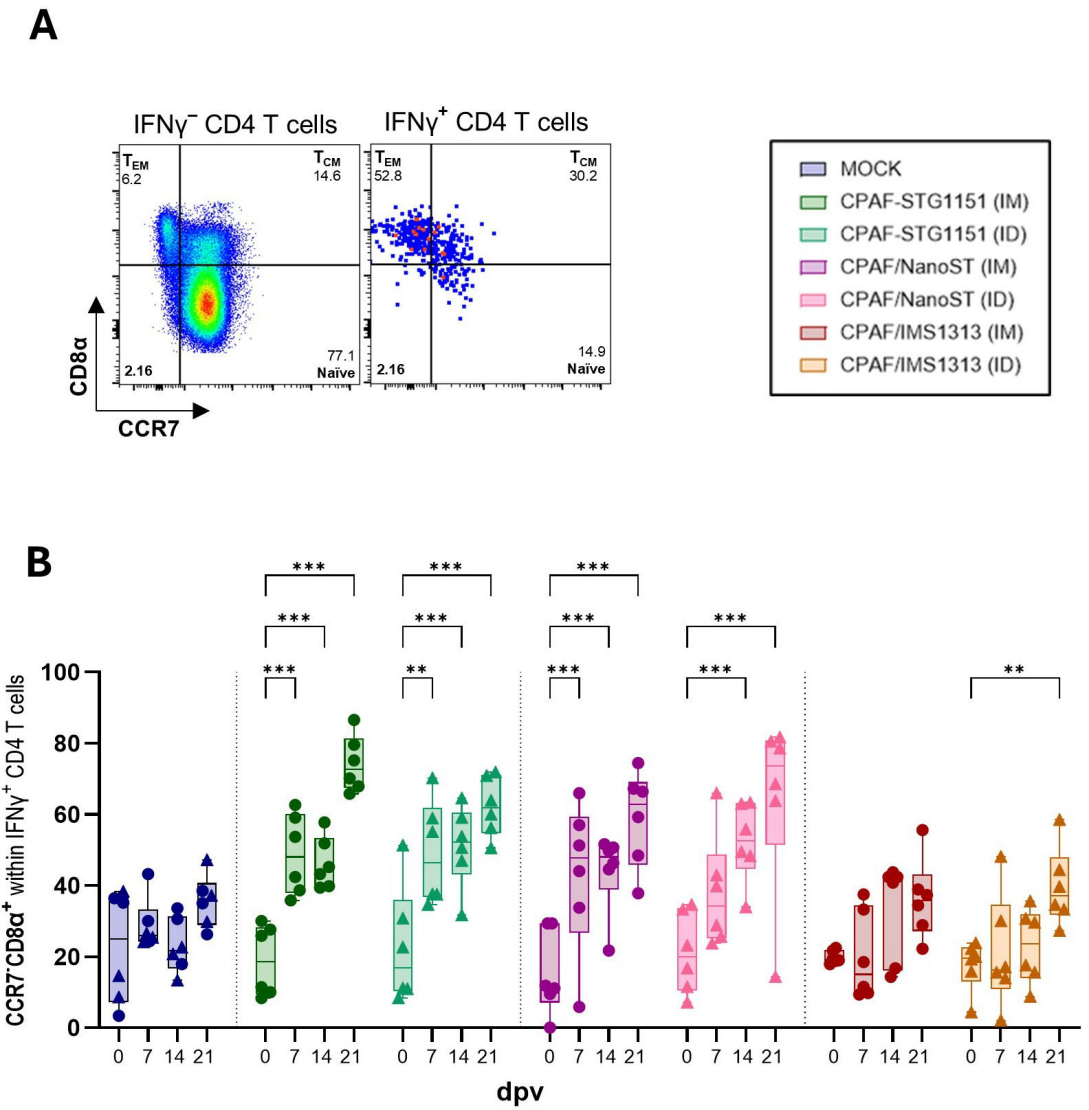
Differentiation of IFNγ^+^ CD4 T cells. Freshly isolated PBMCs were restimulated overnight with CPAF peptide pool and stained for flow cytometry. During data analysis, dead cells and doublets were excluded, as shown in **Supplementary Figure 5A**. After identification of IFNγ^+^ CD4 T cells their differentiation was assessed as shown in **(A)**. Differentiation was analyzed via the CCR7/CD8a expression profile to distinguish naïve (CCR7^+^CD8α^-^), central memory (T_CM_, CCR7^+^CD8α^+^) and effector memory (T_EM_, CCR7^-^CD8α^+^) CD4 T cells. Panel **(B)** shows the frequency of the CCR7^-^CD8α^+^ phenotype within IFNγ producing CD4 T cells over time. Each symbol represents data from one individual pig (n=6 per group). In the MOCK group, three pigs received intramuscular (IM) PBS injections (circle), and three pigs received intradermal (ID) PBS injections (triangle). Statistical analysis was performed via GraphPad using 2-way ANOVA and Tukey multiple comparisons test. The statistical analysis of within-group comparisons are shown. *p < 0.05, **p < 0.01, ***p < 0.001. dpv = days post (first) vaccination.

CC-chemokine receptor 7 (CCR7) is a key lymph node homing receptor expressed on naïve and central memory T cells (50). In pigs, CD4 T cells upregulate CD8α upon antigen encounter, making CD8α expression a marker of antigen experience (51, 52). Taken together, CCR7 and CD8α distinguish porcine naïve T cells (CD8α^−^CCR7^+^), central memory T cells (T_CM_, CD8α^+^CCR7^+^) and effector memory T cells (T_EM_, CD8α^+^CCR7^−^). Therefore, CD4 T cells responding to CPAF restimulation (IFNγ^+^) were further analyzed to determine their differentiation status (**Figure 5A**). Whereas non-responding CD4 T cells predominantly display a naïve phenotype (CD8α^−^CCR7^+^), IFNγ^+^ CD4 T cells are enriched for the T_EM_ phenotype (CD8α^+^CCR7^−^) (**Figure 5A**). In MOCK-vaccinated pigs, the small population of IFNγ^+^ CD4 T cells exhibited a T_EM_ phenotype at relatively low frequencies, with no increase over time (**Figure 5B**). Pigs vaccinated with CPAF-STG1151 or CPAF/NanoST exhibited a clear shift toward an increased T_EM_ phenotype among CPAF-responsive CD4 T cells over time, regardless of vaccine administration route. While the T_EM_ phenotype accounted for only 19% of IFNγ^+^ CD4 T cells on day 0, their proportion increased to 74% by day 21 for pigs vaccinated with the CPAF-STG1151 conjugate (IM) (**Figure 5B**). Vaccination with CPAF/IMS1313 led to a slight increase in the frequency of the T_EM_ phenotype, but overall, it remained similar to what was observed for the MOCK-vaccinated pigs. Together, these findings show that vaccination with CPAF-STG1151 and CPAF/NanoST promoted the differentiation of CPAF-specific CD4 T cells toward a T_EM_ phenotype.

### 3.5 The CPAF-STG1151 conjugate vaccine induced strong systemic anti-CPAF IgG responses

**Figure 6:**
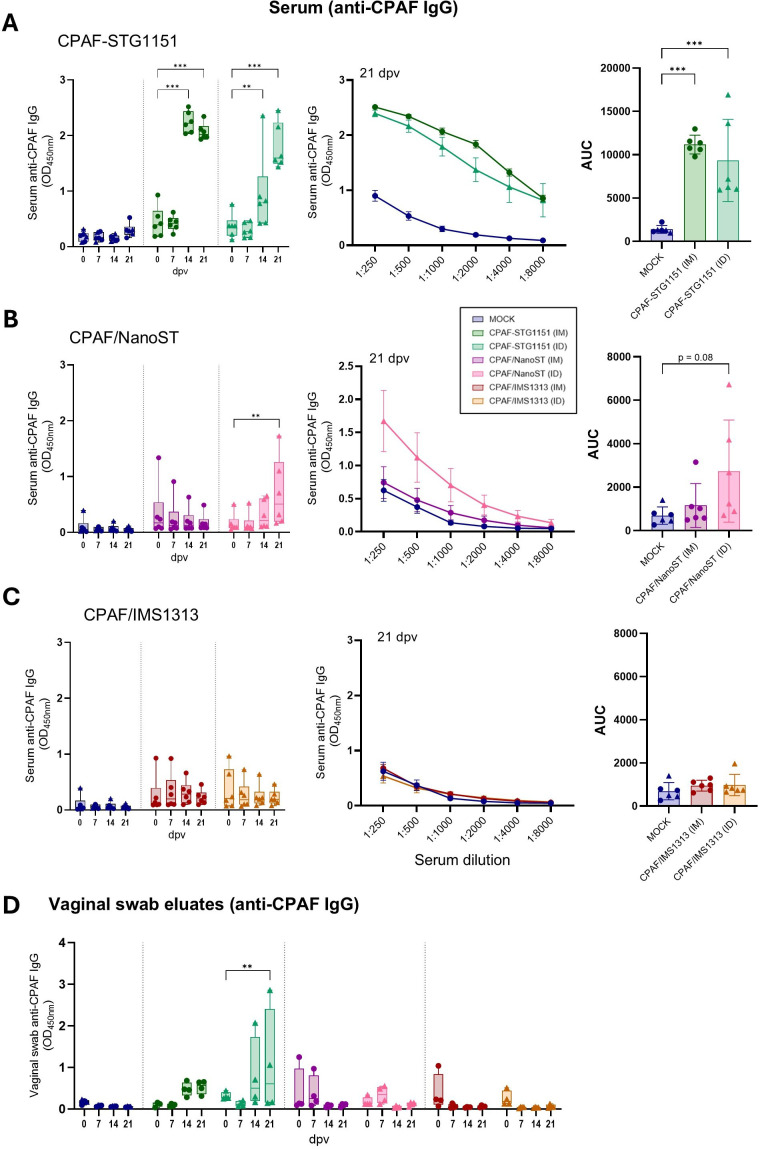
The CPAF-STG1151 vaccine induced a strong systemic CPAF-specific IgG antibody response. Serum samples from CPAF-STG1151 **(A)**, CPAF/NanoST **(B)**, and CPAF/IMS1313 **(C)** vaccinated pigs were analyzed by ELISA for CPAF-specific IgG responses. IgG responses over time are shown on the left (serum dilution 1:1000). Total CPAF-specific IgG in serum samples from 21 dpv is also shown as titration curve with calculated area under the curve (AUC). The data in the titration curve represent the mean ± SEM of 6 pigs per group (middle panel). **(D)** Vaginal swab eluates from CPAF-STG1151, CPAF/NanoST, and CPAF/IMS1313 vaccinated pigs (female) were analyzed by ELISA for CPAF-specific IgG responses. Each symbol represents data from one individual pig (n=6 per group **A-C**, n= 4 per group **D**). In the MOCK group, half of the pigs received intramuscular (IM) PBS injections (circle), and half of the pigs received intradermal (ID) PBS injections (triangle). Statistical analysis was performed via GraphPad using 2-way ANOVA or one-way ANOVA (AUC graph) and Tukey multiple comparisons test. The statistical analysis of within-group comparisons are shown. **p < 0.01, ***p < 0.001. dpv = days post (first) vaccination.

Antibody responses upon vaccination were assessed by ELISA for CPAF-specific IgG in serum and for CPAF-specific IgG and IgA in vaginal swab eluates (**Figure 6**; **Supplementary Figure 8**). While MOCK-vaccinated pigs showed low anti-CPAF IgG levels and no increases over time, CPAF-STG1151 vaccinated animals displayed elevated antibody levels from day 14 onwards (serum dilution 1:1000, **Figure 6A**). By day 14, all IM-vaccinated pigs exhibited high IgG levels with no further increase thereafter. While the response of ID-vaccinated pigs was overall lower than their IM counterparts at 14 dpv, two pigs reached comparable antibody levels 21 dpv. In contrast, for pigs that received the CPAF/NanoST vaccine only the ID administered group exhibited a modest increase in systemic anti-CPAF IgG levels (3/6 pigs); the IM-vaccinated animals in the CPAF/NanoST group showed no detectable IgG above MOCK controls. This was confirmed by serum titration of 21 dpv samples, which showed that even at dilutions as low as 1:250, IM-vaccinated animals did not exhibit higher IgG levels than MOCK controls (**Figure 6B**, center graph). Similarly, vaccination with CPAF/IMS1313 (IM and ID) did not result in measurable anti-CPAF IgG levels in serum and no increase over time was observed (**Figure 6C**). Beyond assessing the systemic IgG response, we examined the presence of anti-CPAF IgG at the mucosal site by performing IgG ELISAs on vaginal swab eluates from female pigs (**Figure 6D**). Notably, the CPAF-STG1151 vaccination led to increased anti-CPAF IgG levels at the vaginal mucosa at 14 and 21 dpv, especially when administered ID (**Figure 6D**). Visualization of individual adaptive immune responses across readouts using a heatmap for the MOCK and CPAF-STG1151 groups revealed that the two pigs in the ID group with elevated serum IgG levels also the ones exhibiting increased anti-CPAF IgG levels at the vaginal mucosa. This might suggest that the IgG detected at the vaginal site is serum-derived rather than locally produced (**Supplementary Figure 9**; pigs #13 and #15). No mucosal presence of anti-CPAF IgG was detected in pigs vaccinated with CPAF/NanoST or CPAF/IMS1313 at either timepoint. In addition to anti-CPAF IgG, anti-CPAF IgA levels were measured in vaginal swab eluates; however, no post-vaccination increase was observed in any experimental group (**Supplementary Figure 8**). Overall, this analysis of humoral responses revealed that only CPAF-STG1151 vaccination consistently induced systemic anti-CPAF IgG with evidence suggesting potential accumulation of IgG at the vaginal mucosa.

## 4 Discussion

We found that the novel conjugate vaccine, CPAF-STG1151, especially administered twice IM, elicited a robust cell-mediated and antibody response in all vaccinated pigs. The T-cell response was primarily characterized by IFNγ, with or without TNFα coproduction, and was mediated by all major T cell subsets (CD4, CD8, γδ T cells). No IL-17A production was observed. Given that excessive Th17 responses to *Chlamydia* have been associated with immunopathology and tissue damage in some models, the absence of a systemic Th17 response may be advantageous (23, 53). However, it is important to note that the lack of a systemic Th17 response does not preclude the presence of a localized mucosal Th17 response. While our previous study also demonstrated induction of Th1 cytokines, IFNγ and TNFα, following vaccination with CPAF and STING pathway agonist ADU-S100 admixture, the responses were lower in magnitude and less consistent (28). This is in line with our parallel studies in mice demonstrating markedly enhanced immune responses after vaccination with CPAF-STG1151 compared to the CPAF/ADU-S100 admixture (Poston et al., manuscript submitted). Moreover, in pigs, the CPAF/ADU-S100 admixture elicited mainly CD4 T cell responses, whereas STING agonist conjugated CPAF-STG1151 engaged all T cell subsets. It should be noted that the absence of a detectable CD8 T cell response in the previous trial may have resulted from using whole protein for restimulation, which relies on cross-presentation by PBMCs and can result in an underestimated CD8 T cell response. Nevertheless, our findings suggest that the CPAF-STG1151 conjugate vaccine may be more effective at engaging CD8 T cells and potentially γδ T cells. The translational relevance of the responses observed in γδ T cells remains unclear, as pigs have a substantially higher frequency of circulating γδ T cells compared to humans (54–56). Enhancing CD8 T cell responses using conjugate vaccines, especially those incorporating TLR ligands, has already been the focus of cancer vaccine research (57, 58). Moreover, a mechanistic study using the model antigen OVA showed that an OVA-TLR7/8 conjugate substantially boosted IFNγ-producing CD4 and CD8 T cells relative to OVA with free TLR7/8 agonist (59). While conjugates of STING agonists with full-length proteins have not been studied thus far, peptide antigens linked to a STING agonist have been shown to elicit strong CD8 T cell responses (40). Thus, it seems likely that the conjugation of the STING agonist STG1151 to the protein antigen CPAF enhances cross-presentation to CD8 T cells resulting in a more balanced CD4/CD8 response. While IFNγ-secreting CD4 T cells have been identified as critical mediators of protective immunity against *Ct*, the understanding of the contribution of CD8 T cells remains incomplete. Some studies suggest that CD8 T cells are dispensable, whereas others support their contribution to protective immunity particularly via IFNγ production rather than classical cytotoxicity (60). For example, depletion of CD8 T cells in B-cell-deficient mice had no effect on the course of a secondary infection; in contrast, CD4-depleted B-cell-deficient mice failed to control *Ct* infection (61). However, adoptive transfer experiments using *Ct*-specific IFNγ-producing CD8 T cell clones demonstrated that these cells conferred at least partial protection (62–64). Moreover, a trachoma vaccine study in macaques showed that depletion of CD8 T cells in previously protected animals significantly reduced protection upon challenge, as evidenced by an increased bacterial burden and prolonged *Ct* shedding (65). In humans, the presence or frequency of *Ct*-specific CD8 T cells or CD8 TEM subsets do not appear to correlate with reinfection status (19, 20, 66). However, *Ct*-specific IFNγ-producing CD8 T cells have been linked to lower bacterial loads in reinfected women and showed a slight association with reduced ascension of infection (19, 66). These findings indicate that while *Ct*-specific CD8 T cells have the potential to confer protection, they are likely not robustly induced during natural infection. Whether vaccination-induced CD8 T cells enhance protection and promote bacterial clearance remains to be determined.

Besides the induction of strong systemic Th1 responses, the CPAF-STG1151 vaccination resulted in the differentiation of CPAF-specific CD4 T cells into an effector memory phenotype (CCR7^-^CD8α^+^). By day 21 post-vaccination with CPAF-STG1151 (IM) over 65% of IFNγ-producing CD4 T cells displayed a T_EM_ phenotype compared to 10-30% prior to vaccination indicating a marked expansion of systemic effector memory populations with possible mucosal homing capacity. However, the interpretation of a durable memory component should be made with caution, given the relatively short study duration. Parabiosis studies in mice have demonstrated that optimal *Ct* clearance after vaccination depends on both uterine tissue-resident memory T cells (T_RM_) and the recruitment of circulating memory T cells (67). Although T_RM_ subsets were not assessed in our study, the observed differentiation of circulating CD4 T cells may reflect the early establishment of functional memory.

No significant differences were observed between IM and ID administration routes with respect to the induction of systemic Th1 responses, generation of CPAF-specific effector memory T cells, or humoral immune responses. Surprisingly, a trend toward lower frequency of multifunctional CD4 T cells (IFNγ^+^TNFα^+^) and a delayed antibody response in the CPAF-STG1151 ID group compared to the IM group was noted. In addition, while responses in the IM group were quite consistent, the responses in the ID group were also largely driven by 2–3 responder animals (**Supplementary Figure 9**) indicating biological factors or variability in the injection technique. Although a needle-free intradermal injection device previously shown to work in pigs was used and no leaks or other issues were observed, some variability in depth of vaccine administration cannot be entirely ruled out. ID vaccination is generally thought to elicit immune responses comparable or greater than those achieved by IM administration, often requiring only 10-40% of the vaccine dose (68). However, this can vary depending on the vaccine formulation and platform used (69). Evidence related to ID administration of conjugate vaccines remains limited. A study evaluating the pneumococcal-diphtheria-toxin conjugate vaccine (PCV13) in mice found that ID delivery induced weaker humoral responses compared to IM administration suggesting that conjugate vaccines may be less effective when delivered via the ID route (70). However, the exact reasons why ID administration did not outperform IM for CPAF-STG1151 remain unclear but may reflect a combination of biological factors, injection technique and vaccine composition.

While the CPAF-STG1151 vaccine was immunogenic, CPAF formulated in Montanide™ IMS 1313 N VG PR did not induce a measurable immune response in our setting. This adjuvant is a ready-to-dilute microemulsion (oil-in-water) and designed for use in poultry and swine vaccines. The observation that this adjuvant is not or is only slightly immunogenic when used in subunit vaccines is consistent with previous studies in pigs (71–74). In a subunit vaccine against *Lawsonia intracellularis*, IMS 1313 adjuvant resulted in low or undetectable serum IgG titers, particularly when compared with water-in-oil emulsions, and did not induce an IFNγ response (73).

Besides conjugating CPAF to the STING adjuvant, a nanoparticle-based vaccine formulation was included in our study to minimize the systemic diffusion of STING pathway agonists. The full-length protein CPAF from *Ct* was adsorbed onto positively charged corn-derived nanoparticles (Nano-11) together with the synthetic cyclic dinucleotide ADU-S100 (STING agonist). Surprisingly, the nanoparticle-based vaccine elicited only a weak cell-mediated immune response, particularly when compared to the response observed in CPAF-STG1151 vaccinated animals. Nevertheless, a slight increase of IFNγ^+^ and IFNγ^+^TNFα^+^ PBMCs was observed 14 dpv and this CPAF-specific cytokine production was mediated by all T cell subsets. Despite their low frequency, CPAF-specific CD4 T cells from CPAF/NanoST vaccinated pigs differentiated over time into an effector memory phenotype indicating vaccine mediated memory development. Notably, compared to IM administration, cytokine responses by PBMCs were slightly more robust following ID administration. This trend was also reflected in the systemic humoral response: while IM-vaccinated pigs failed to develop a measurable IgG response, 3 out of 6 ID-vaccinated pigs showed increasing anti-CPAF IgG titers over time. Overall, the responses elicited by the CPAF/NanoST vaccine candidate were consistently lower than those induced by CPAF-STG1151. This is particularly striking considering that the CPAF-STG1151 vaccine contained only 0.4 nmol of STING agonist per dose, compared with about 68 nmol of ADU-S100 in CPAF/NanoST. However, when comparing CPAF-STG1151 and CPAF/NanoST, it is important to consider that the STING agonists differ between the two vaccine formulations, as do their delivery systems and, consequently, their mechanisms of intracellular access. Hence, the significantly lower response in the CPAF/NanoST group might not be due to the nanoparticle platform but a consequence of potency differences of the STING agonists used (29, 75). While the CPAF conjugate vaccine incorporated STG1151, a novel conjugatable STING agonist synthesized by InvivoGen, the CPAF/NanoST vaccine utilized ADU-S100. Both are synthetic cyclic dinucleotides, but they differ in structure. According to *in vitro* studies by InvivoGen, STG1151 alone demonstrates potency comparable to ADU-S100 in activating the STING pathway. However, when conjugated to CPAF, STG1151 exhibits a two-log increase in potency in J774-Dual™ cells (a murine macrophage cell line). This increase may be due to favorable phagocytosis. Moreover, most STING agonists have only been evaluated for their ability to bind and activate human and mouse STING receptors, with limited data available for pigs. Hernandez and colleagues (42) characterized the response of porcine monocyte-derived DCs to c-di-AMP, which has lower affinity to STING than ADU-S100, and found that it induced TNFα and IL-1β production, albeit at lower levels than those observed in mouse bone marrow–derived DCs (42). Consistent with this observation, Cong et al. (76) found that porcine STING binds c-di-GMP and c-di-AMP with low affinity, which also appears lower than affinities reported for human and murine STING (76–79). To date, no comprehensive analysis has been conducted on the affinity and potency of ADU-S100 or STG1151 in porcine cells, preventing definitive conclusions about why the CPAF/NanoST formulation failed to elicit adaptive immune responses. However, it seems more likely that this was due to the suboptimal engagement of porcine STING by ADU-S100 rather than failure of the delivery platform itself. In mice, ID vaccination against OVA using the combination adjuvant NanoST (Nano11 + ADU-S100) resulted in increased frequencies of OVA-specific Th1 CD4 T cells and anti-OVA serum IgG titers compared to either adjuvant alone, highlighting both the contribution of ADU-S100 and the general suitability of the nanoparticle platform (80). However, in pigs, the combination of Nano11 and c-di-AMP, the natural analog of ADU-S100, did not significantly enhance anti-OVA IgG titers compared to Nano11 alone, calling into question the *in vivo* adjuvant activity of c-di-AMP in pigs (42). Similarly, pigs vaccinated ID/IM against IAV with either Nano11 or Nano11 + ADU-S100 showed only minor differences in T cell responses and antibody levels in the lung and serum between the two formulations, indicating only a limited effect of ADU-S100 (43). Even without a substantial contribution of ADU-S100, some level of adaptive immune response would still be expected, as NanoST has been shown to induce at least humoral immune responses via ID and IM routes in swine influenza vaccines (43, 44). Hernandez et al. (44) demonstrated that ID delivery of NanoST with whole inactivated IAV resulted in enhanced humoral immune responses compared to IM administration in pigs (44). However, IM vaccination still resulted in detectable IgG and IgA titers, in contrast to the complete lack of antibody responses observed after IM administration in our study. This difference may be explained by the fact that the whole inactivated IAV vaccine contained multiple viral proteins and possibly PAMPs, whereas our subunit vaccine included only a single protein and likely provided less innate immune stimulation. Consistent with this, IAV peptides adsorbed onto Nano-11 with Poly(I:C) failed to elicit substantial antibody responses, whereas the whole inactivated virus preparation induced local IgA and IgG titers highlighting the impact of antigen composition (81). Future studies should focus on a detailed evaluation of phytoglycogen nanoparticles in single-protein formulations, along with assessments of ADU-S100 potency on porcine STING, to fully determine its suitability as an adjuvant in pigs.

Overall, our study successfully identified a vaccine formulation, namely CPAF-STG1151, capable of inducing a robust systemic immune response. However, several limitations should be considered when interpreting the results. First, the study duration was limited to 21 days post (first) vaccination, capturing early effector responses but precluding evaluation of long-term memory. Second, the interval between the prime and boost was limited to 7 days, which is shorter than the commonly used 21–28 day interval that supports memory formation and more robust secondary immune responses. This short interval was chosen for consistency with our previous work which used this setup due to logistical constraints (28, 49). While some boosting likely occurred with this accelerated schedule, we did not include a single-dose control group and therefore cannot fully assess the impact of the early boost at 7 days. Future studies will evaluate the CPAF-STG1151 vaccine with an extended prime – boost interval of 21 days.

While the primary goal of this study was to identify a vaccine formulation capable of eliciting a robust Th1-biased systemic immune response, this represents only the first step in vaccine optimization against *Ct*. Protective immunity will require both circulating memory T cells and tissue-resident memory T cells (TRMs) at the site of infection, as demonstrated in parabiosis experiments and humanized mouse models (67). Critically, induction of TRMs and protection in these models depended on mucosal vaccination strategies, such as IN delivery (67). Hence, the absence of an IN administration in the present study represents a limitation that will be addressed in a follow-up study with CPAF-STG1151 focused on mucosal immunization. Additionally, in our study, mucosal sampling was restricted to female animals (vaginal swabs), limiting the evaluation of local immune responses in males. Moreover, immune responses were assessed exclusively against the vaccine antigen, without evaluation of responses to native chlamydial antigens (whole *Ct* or *Cs*). Therefore, the extent to which these responses translate into pathogen recognition and protective efficacy remains to be determined. While *Ct* vaccine efficacy can, in principle, be evaluated using a transcervical challenge model in pigs, this approach has some limitations. Notably, *Ct* is cleared rapidly, often within days, making it difficult to reliably detect vaccine effects based on bacterial burden (14, 28, 82). In addition, the rather inconsistent occurrence of clinical signs, histopathological lesions or vaginal discharge complicates the assessment of successful infection (14, 82). Alternative approaches, such as modifying the challenge site or inclusion of additional challenge models, such as guinea pigs, could strengthen the evaluation of vaccine efficacy. Regardless of the model used, future challenge studies should not only assess reductions in bacterial burden and pathology but also place particular emphasis on possible mucosal correlates of protection, including TRMs and local IgA responses.

Despite these limitations, our study provides valuable insights into systemic immunogenicity and informs the design of future trials aimed at optimizing both systemic and mucosal responses. In summary, our work showed that while the CPAF/IMS1313 vaccine failed to induce an adaptive immune response, the CPAF/NanoST vaccine induced low-level T-cell and antibody responses with the needle-free ID administration outperforming the IM administration. The most immunogenic vaccine candidate however was CPAF-STG1151: this conjugate vaccine elicited a strong systemic Th1 response, differentiation of CD4 T cells into effector memory phenotypes and high serum levels of anti-CPAF IgG and detectable anti-CPAF IgG in vaginal secretions. These results highlight CPAF-STG1151 as a highly immunogenic *Ct* vaccine candidate in the pig model.

## Data Availability

The original contributions presented in the study are included in the article/**Supplementary Material**. Further inquiries can be directed to the corresponding author.

## References

[1] den Heijer CDJ Hoebe CJPA Driessen JHM Wolffs P van den Broek IVF Hoenderboom BM . Chlamydia trachomatis and the risk of pelvic inflammatory disease, ectopic pregnancy, and female infertility: A retrospective cohort study among primary care patients. Clin Infect Dis. (2019) 69:1517–25. doi: 10.1093/cid/ciz429. PMID: 31504315 PMC6792126

[2] Hillier SL Bernstein KT Aral S . A review of the challenges and complexities in the diagnosis, etiology, epidemiology, and pathogenesis of pelvic inflammatory disease. J Infect Dis. (2021) 224:S23–8. doi: 10.1093/infdis/jiab116. PMID: 34396398 PMC8365114

[3] Abraham S Juel HB Bang P Cheeseman HM Dohn RB Cole T . Safety and immunogenicity of the chlamydia vaccine candidate CTH522 adjuvanted with CAF01 liposomes or aluminium hydroxide: A first-in-human, randomised, double-blind, placebo-controlled, phase 1 trial. Lancet Infect Dis. (2019) 19:1091–100. doi: 10.1016/S1473-3099(19)30279-8. PMID: 31416692

[4] Pollock KM Borges ÁH Cheeseman HM Rosenkrands I Schmidt KL Søndergaard RE . An investigation of trachoma vaccine regimens by the chlamydia vaccine CTH522 administered with cationic liposomes in healthy adults (CHLM-02): A phase 1, double-blind trial. Lancet Infect Dis. (2024) 24:829–44. doi: 10.1016/S1473-3099(24)00147-6. PMID: 38615673

[5] Käser T . Swine as biomedical animal model for T-cell research—Success and potential for transmittable and non-transmittable human diseases. Mol Immunol. (2021) 135:95–115. doi: 10.1016/j.molimm.2021.04.004. PMID: 33873098

[6] Pabst R . The pig as a model for immunology research. Cell Tissue Res. (2020) 380:287–304. doi: 10.1007/s00441-020-03206-9. PMID: 32356014 PMC7223737

[7] Lunney JK Van Goor A Walker KE Hailstock T Franklin J Dai C . Importance of the pig as a human biomedical model. Sci Transl Med. (2021) 13:eabd5758. doi: 10.1126/scitranslmed.abd5758. PMID: 34818055

[8] Dimond ZE Hefty PS . Comprehensive genome analysis and comparisons of the swine pathogen, Chlamydia suis reveals unique ORFs and candidate host-specificity factors. Pathog Dis. (2020) 79:ftaa035. doi: 10.1093/femspd/ftaa035. PMID: 32639528 PMC7948067

[9] Hoffmann K Schott F Donati M Francesco D Hässig M Wanninger S . Prevalence of chlamydial infections in fattening pigs and their influencing factors. PloS One. (2015) 10:e0143576. doi: 10.1371/journal.pone.0143576. PMID: 26619187 PMC4664257

[10] Li M Jelocnik M Yang F Gong J Kaltenboeck B Polkinghorne A . Asymptomatic infections with highly polymorphic Chlamydia suis are ubiquitous in pigs. BMC Vet Res. (2017) 13:370. doi: 10.1186/s12917-017-1295-x. PMID: 29191191 PMC5710075

[11] Häcker G . Chlamydia in pigs: Intriguing bacteria associated with sub-clinical carriage and clinical disease, and with zoonotic potential. Front Cell Dev Biol. (2024) 12:1301892. doi: 10.3389/fcell.2024.1301892. PMID: 39206090 PMC11349706

[12] Schautteet K Vanrompay D . Chlamydiaceae infections in pig. Vet Res. (2011) 42:29. doi: 10.1186/1297-9716-42-29. PMID: 21314912 PMC3041669

[13] Amaral AF Rahman KS Kick AR Cortes LM Robertson J Kaltenboeck B . Mucosal vaccination with UV-inactivated Chlamydia suis in pre-exposed outbred pigs decreases pathogen load and induces CD4 T-cell maturation into IFN-γ+ effector memory cells. Vaccines. (2020) 8:3. doi: 10.3390/vaccines8030353. PMID: 32630694 PMC7564508

[14] Käser T Pasternak JA Delgado-Ortega M Hamonic G Lai K Erickson J . Chlamydia suis and Chlamydia trachomatis induce multifunctional CD4 T cells in pigs. Vaccine. (2017) 35:91–100. doi: 10.1016/j.vaccine.2016.11.050. PMID: 27894718

[15] Gondek DC Olive AJ Stary G Starnbach MN . CD4+ T cells are necessary and sufficient to confer protection against C. trachomatis infection in the murine upper genital tract. J Immunol. (2012) 189:2441–9. doi: 10.4049/jimmunol.1103032. PMID: 22855710 PMC3690950

[16] Helble JD Gonzalez RJ von Andrian UH Starnbach MN . Gamma interferon is required for Chlamydia clearance but is dispensable for T cell homing to the genital tract. mBio. (2020) 11:10.1128/mbio.00191–20. doi: 10.1128/mbio.00191-20. PMID: 32184237 PMC7078466

[17] Olivas J Nogueira C Helble J Starnbach MN . Cytotoxic CD4+ T cells are induced during infection with Chlamydia trachomatis. J Immunol. (2024) 213:328–38. doi: 10.4049/jimmunol.2300131. PMID: 38905023 PMC11279525

[18] Bakshi RK Gupta K Jordan SJ Chi X Lensing SY Press CG . An adaptive Chlamydia trachomatis-specific IFN-γ-producing CD4+ T cell response is associated with protection against Chlamydia reinfection in women. Front Immunol. (2018) 9:1981. doi: 10.3389/fimmu.2018.01981. PMID: 30245688 PMC6137090

[19] Russell AN Zheng X O’Connell CM Wiesenfeld HC Hillier SL Taylor BD . Identification of Chlamydia trachomatis antigens recognized by T cells from highly exposed women who limit or resist genital tract infection. J Infect Dis. (2016) 214:1884–92. doi: 10.1093/infdis/jiw485. PMID: 27738051 PMC5142095

[20] Yount KS Chen CJ Kollipara A Liu C Mokashi NV Zheng X . T cell signatures associated with reduced Chlamydia trachomatis reinfection in a highly exposed cohort. JCI Insight. (2025) 10:e189388. doi: 10.1172/jci.insight.189388. PMID: 40014387 PMC11991011

[21] Rixon JA Depew CE McSorley SJ . Th1 cells are dispensable for primary clearance of Chlamydia from the female reproductive tract of mice. PloS Pathog. (2022) 18:e1010333. doi: 10.1371/journal.ppat.1010333. PMID: 35196366 PMC8901068

[22] Rixon JA Fong KD Morris C Nguyen AT Depew CE McSorley SJ . Elimination of Chlamydia muridarum from the female reproductive tract is IL-12p40 dependent, but independent of Th1 and Th2 cells. PloS Pathog. (2024) 20:e1011914. doi: 10.1371/journal.ppat.1011914. PMID: 38166152 PMC10786385

[23] Chen Y Chen J Xu L Tang M Wang C . IL-17 and Th17 cells in Chlamydiae infection. Immunology. (2026) 177:234–46. doi: 10.1111/imm.70069. PMID: 41309017

[24] Scurlock AM Frazer LC Andrews CW O’Connell CM Foote IP Bailey SL . Interleukin-17 contributes to generation of Th1 immunity and neutrophil recruitment during Chlamydia muridarum genital tract infection but is not required for macrophage influx or normal resolution of infection. Infect Immun. (2011) 79:1349–62. doi: 10.1128/IAI.00984-10. PMID: 21149587 PMC3067500

[25] Li Y Warren JA Poston TB Clutton G Shaw FR Conrad SZ . Low-frequency, sustained CD4 T-cell responses Chlamydia trachomatis in women: Predominant targeting of chlamydial proteaselike activity factor (CPAF). J Infect Dis. (2024) 231(2):e385–e395. doi: 10.1093/infdis/jiae443. PMID: 39250505 PMC11841636

[26] Snavely EA Kokes M Dunn JD Saka HA Nguyen BD Bastidas RJ . Reassessing the role of the secreted protease CPAF in Chlamydia trachomatis infection through genetic approaches. Pathog Dis. (2014) 71:336–51. doi: 10.1111/2049-632X.12179. PMID: 24838663 PMC4270368

[27] Rajeeve K Das S Prusty BK Rudel T . Chlamydia trachomatis paralyses neutrophils to evade the host innate immune response. Nat Microbiol. (2018) 3:824–35. doi: 10.1038/s41564-018-0182-y. PMID: 29946164

[28] Bettin L Stadler M Unterweger C Dippel M Harris JM Buzanich-Ladinig A . A cyclic-di-AMP adjuvanted CPAF protein vaccine is immunogenic in swine, but it fails to reduce genital Chlamydia trachomatis burden. Vaccines. (2025) 13:468. doi: 10.3390/vaccines13050468. PMID: 40432080 PMC12115861

[29] Van Herck S Feng B Tang L . Delivery of STING agonists for adjuvanting subunit vaccines. Adv Drug Delivery Rev. (2021) 179:114020. doi: 10.1016/j.addr.2021.114020. PMID: 34756942

[30] Volckmar J Knop L Stegemann-Koniszewski S Schulze K Ebensen T Guzmán CA . The STING activator c-di-AMP exerts superior adjuvant properties than the formulation poly(I:C)/CpG after subcutaneous vaccination with soluble protein antigen or DEC-205-mediated antigen targeting to dendritic cells. Vaccine. (2019) 37:4963–74. doi: 10.1016/j.vaccine.2019.07.019. PMID: 31320219

[31] Van Dis E Sogi KM Rae CS Sivick KE Surh NH Leong ML . STING-activating adjuvants elicit a Th17 immune response and protect against Mycobacterium tuberculosis infection. Cell Rep. (2018) 23:1435–47. doi: 10.1016/j.celrep.2018.04.003. PMID: 29719256 PMC6003617

[32] Jong RM Van Dis E Berry SB Nguyenla X Baltodano A Pastenkos G . Mucosal vaccination with cyclic dinucleotide adjuvants induces effective T cell homing and IL-17–dependent protection against Mycobacterium tuberculosis infection. J Immunol. (2022) 208:407–19. doi: 10.4049/jimmunol.2100029. PMID: 34965963 PMC8755605

[33] Poston TB Girardi J Kim M Zwarycz P Polson AG Yount KS . Intranasal immunization with CPAF combined with ADU-S100 induces an effector CD4 T cell response and reduces bacterial burden following intravaginal infection with Chlamydia muridarum. Vaccine. (2025) 43:126526. doi: 10.1016/j.vaccine.2024.126526. PMID: 39536454 PMC11817958

[34] Wang J Li P Wu MX . Natural STING agonist as an “ideal” adjuvant for cutaneous vaccination. J Invest Dermatol. (2016) 136:2183–91. doi: 10.1016/j.jid.2016.05.105. PMID: 27287182 PMC6091668

[35] Shen Y Huang W Nie J Zhang L . Progress update on STING agonists as vaccine adjuvants. Vaccines. (2025) 13:371. doi: 10.3390/vaccines13040371. PMID: 40333245 PMC12030840

[36] Gehrcken L Deben C Smits E Van Audenaerde JRM . STING agonists and how to reach their full potential in cancer immunotherapy. Adv Sci (Weinh). (2025) 12:2500296. doi: 10.1002/advs.202500296. PMID: 40145387 PMC12061341

[37] Xu Z Moyle PM . Bioconjugation approaches to producing subunit vaccines composed of protein or peptide antigens and covalently attached toll-like receptor ligands. Bioconjug Chem. (2018) 29:572–86. doi: 10.1021/acs.bioconjchem.7b00478. PMID: 28891637

[38] Manabe Y Fukase K . Innovative vaccine strategy: self-adjuvanting conjugatevaccines. In: Kabayama K Inokuchi J (eds). Glycolipids. Methods in Molecular Biology, vol 2613. Springer US, New York, NY (2023). p. 55–72. doi: 10.1007/978-1-0716-2910-9_5, PMID: 36587070

[39] Hanna CC Ashhurst AS Quan D Maxwell JWC Britton WJ Payne RJ . Synthetic protein conjugate vaccines provide protection against Mycobacterium tuberculosis in mice. Proc Natl Acad Sci. (2021) 118:e2013730118. doi: 10.1073/pnas.2013730118. PMID: 33468674 PMC7848748

[40] Wang X Huang Z Xing L Shang L Jiang J Deng C . STING agonist-based ER-targeting molecules boost antigen cross-presentation. Nature. (2025) 641:202–10. doi: 10.1038/s41586-025-08758-w. PMID: 40140567 PMC12043507

[41] Zhang M Ji Y Liu M Dai Y Zhang H Tong S . Nano-delivery of STING agonists: Unraveling the potential of immunotherapy. Acta Biomater. (2025) 197:104–20. doi: 10.1016/j.actbio.2025.03.054. PMID: 40164370

[42] Hernandez-Franco JF Mosley YYC Franco J Ragland D Yao Y HogenEsch H . Effective and safe stimulation of humoral and cell-mediated immunity by intradermal immunization with a cyclic dinucleotide/nanoparticle combination adjuvant. J Immunol. (2021) 206:700–11. doi: 10.4049/jimmunol.2000703. PMID: 33380496

[43] Patil V Hernandez-Franco JF Yadagiri G Bugybayeva D Dolatyabi S Feliciano-Ruiz N . A split influenza vaccine formulated with a combination adjuvant composed of alpha-d-glucan nanoparticles and a STING agonist elicits cross-protective immunity in pigs. J Nanobiotechnol. (2022) 20:477. doi: 10.1186/s12951-022-01677-2. PMID: 36369044 PMC9652892

[44] Hernandez-Franco JF Yadagiri G Patil V Bugybayeva D Dolatyabi S Dumkliang E . Intradermal vaccination against influenza with a STING-targeted nanoparticle combination adjuvant induces superior cross-protective humoral immunity in swine compared with intranasal and intramuscular immunization. Vaccines. (2023) 11:1699. doi: 10.3390/vaccines11111699. PMID: 38006031 PMC10675188

[45] Criscuolo E Caputo V Diotti RA Sautto GA Kirchenbaum GA Clementi N . Alternative methods of vaccine delivery: An overview of edible and intradermal vaccines. J Immunol Res. (2019) 2019:8303648. doi: 10.1155/2019/8303648. PMID: 30949518 PMC6425294

[46] Ledesma-Feliciano C Chapman R Hooper JW Elma K Zehrung D Brennan MB . Improved DNA vaccine delivery with needle-free injection systems. Vaccines (Basel). (2023) 11:280. doi: 10.3390/vaccines11020280. PMID: 36851159 PMC9964240

[47] Pantchev A Sting R Bauerfeind R Tyczka J Sachse K . Detection of all Chlamydophila and Chlamydia spp. of veterinary interest using species-specific real-time PCR assays. Comp Immunol Microbiol Infect Dis. (2010) 33:473–84. doi: 10.1016/j.cimid.2009.08.002. PMID: 19733907

[48] Lu F Mencia A Bi L Taylor A Yao Y HogenEsch H . Dendrimer-like alpha-d-glucan nanoparticles activate dendritic cells and are effective vaccine adjuvants. J Control Release. (2015) 204:51–9. doi: 10.1016/j.jconrel.2015.03.002. PMID: 25747143

[49] Proctor J Stadler M Cortes LM Brodsky D Poisson L Gerdts V . A TriAdj-adjuvanted Chlamydia trachomatis CPAF protein vaccine is highly immunogenic in pigs. Vaccines. (2024) 12:4. doi: 10.3390/vaccines12040423. PMID: 38675805 PMC11054031

[50] Förster R Davalos-Misslitz AC Rot A . CCR7 and its ligands: Balancing immunity and tolerance. Nat Rev Immunol. (2008) 8:362–71. doi: 10.1038/nri2297. PMID: 18379575

[51] Saalmüller A Werner T Fachinger V . T-helper cells from naive to committed. Vet Immunol Immunopathol. (2002) 87:137–45. doi: 10.1016/S0165-2427(02)00045-4. PMID: 12072228

[52] Gerner W Käser T Saalmüller A . Porcine T lymphocytes and NK cells--an update. Dev Comp Immunol. (2009) 33:310–20. doi: 10.1016/j.dci.2008.06.003. PMID: 18601948

[53] Dockterman J Coers J . Immunopathogenesis of genital Chlamydia infection: insights from mouse models. Pathog Dis. (2021) 79:ftab012. doi: 10.1093/femspd/ftab012. PMID: 33538819 PMC8189015

[54] Fonseca S Pereira V Lau C Teixeira MA Bini-Antunes M Lima M . Human peripheral blood gamma delta T cells: report on a series of healthy Caucasian Portuguese adults and comprehensive review of the literature. Cells. (2020) 9:729. doi: 10.3390/cells9030729. PMID: 32188103 PMC7140678

[55] Sedlak C Patzl M Saalmüller A Gerner W . IL-12 and IL-18 induce interferon-γ production and de novo CD2 expression in porcine γδ T cells. Dev Comp Immunol. (2014) 47:115–22. doi: 10.1016/j.dci.2014.07.007. PMID: 25036760

[56] Bettin L Darbellay J van Kessel J Buchanan R Popowych Y Gerdts V . Co-stimulation by TLR7/8 ligand R848 modulates IFN-γ production of porcine γδ T cells in a microenvironment-dependent manner. Dev Comp Immunol. (2023) 138:104543. doi: 10.1016/j.dci.2022.104543. PMID: 36130633

[57] Zom GG Welters MJP Loof NM Goedemans R Lougheed S Valentijn RRPM . TLR2 ligand-synthetic long peptide conjugates effectively stimulate tumor-draining lymph node T cells of cervical cancer patients. Oncotarget. (2016) 7:67087–100. doi: 10.18632/oncotarget.11512. PMID: 27564262 PMC5341859

[58] Lynn GM Sedlik C Baharom F Zhu Y Ramirez-Valdez RA Coble VL . Peptide–TLR-7/8a conjugate vaccines chemically programmed for nanoparticle self-assembly enhance CD8 T-cell immunity to tumor antigens. Nat Biotechnol. (2020) 38:320–32. doi: 10.1038/s41587-019-0390-x. PMID: 31932728 PMC7065950

[59] Kastenmüller K Wille-Reece U Lindsay RWB Trager LR Darrah PA Flynn BJ . Protective T cell immunity in mice following protein-TLR7/8 agonist-conjugate immunization requires aggregation, type I IFN, and multiple DC subsets. J Clin Invest. (2011) 121:1782–96. doi: 10.1172/JCI45416. PMID: 21540549 PMC3083762

[60] Wizel B Nyström-Asklin J Cortes C Tvinnereim A . Role of CD8+ T cells in the host response to Chlamydia. Microbes Infect. (2008) 10:1420–30. doi: 10.1016/j.micinf.2008.08.006. PMID: 18790073 PMC2640455

[61] Morrison SG Su H Caldwell HD Morrison RP . Immunity to murine Chlamydia trachomatis genital tract reinfection involves B cells and CD4+ T cells but not CD8+ T cells. Infect Immun. (2000) 68:6979–87. doi: 10.1128/iai.68.12.6979-6987.2000. PMID: 11083822 PMC97807

[62] Igietseme JU Magee DM Williams DM Rank RG . Role for CD8+ T cells in antichlamydial immunity defined by Chlamydia-specific T-lymphocyte clones. Infect Immun. (1994) 62:5195–7. doi: 10.1128/iai.62.11.5195-5197.1994. PMID: 7927806 PMC303248

[63] Lampe MF Wilson CB Bevan MJ Starnbach MN . Gamma interferon production by cytotoxic T lymphocytes is required for resolution of Chlamydia trachomatis infection. Infect Immun. (1998) 66:5457–61. doi: 10.1128/iai.66.11.5457-5461.1998. PMID: 9784557 PMC108683

[64] Starnbach MN Bevan MJ Lampe MF . Protective cytotoxic T lymphocytes are induced during murine infection with Chlamydia trachomatis. J Immunol. (1994) 153:5183–9. doi: 10.4049/jimmunol.153.11.5183. PMID: 7525725

[65] Olivares-Zavaleta N Whitmire WM Kari L Sturdevant GL Caldwell HD . CD8+ T cells define an unexpected role in live-attenuated vaccine protective immunity against Chlamydia trachomatis infection. J Immunol. (2014) 192:4648–54. doi: 10.4049/jimmunol.1400120. PMID: 24711617 PMC4023123

[66] Geisler WM Legg SB Moylan DC Gupta K Van Der Pol B Tiwari H . Chlamydia trachomatis-specific interferon-γ-producing CD8 T-cells are associated with lower chlamydia bacterial load in reinfected women. Immunohorizons. (2025) 9:vlaf004. doi: 10.1093/immhor/vlaf004. PMID: 40165713 PMC11959114

[67] Stary G Olive A Radovic-Moreno AF Gondek D Alvarez D Basto PA . Vaccines. A mucosal vaccine against Chlamydia trachomatis generates two waves of protective memory T cells. Science. (2015) 348:aaa8205. doi: 10.1126/science.aaa8205. PMID: 26089520 PMC4605428

[68] Zhang L Wang W Wang S . Effect of vaccine administration modality on immunogenicity and efficacy. Expert Rev Vaccines. (2015) 14:1509–23. doi: 10.1586/14760584.2015.1081067. PMID: 26313239 PMC4915566

[69] Hickling J Jones K Friede M Zehrung D Chen D Kristensen D . Intradermal delivery of vaccines: potential benefits and current challenges. Bull World Health Organ. (2011) 89:221–6. doi: 10.2471/BLT.10.079426. PMID: 21379418 PMC3044245

[70] Uijen RF van Beek LF van Opzeeland F Simonetti E van Selm S Bonduelle O . Intradermal administration of the pneumococcal conjugate vaccine in mice results in lower antibody responses as compared to intramuscular administration. Vaccine. (2023) 41:10–4. doi: 10.1016/j.vaccine.2022.11.043. PMID: 36446656

[71] Hwang J Lee KN Kim SM Kim H Park SH Kim DW . Enhanced effects of ISA 207 adjuvant via intradermal route in foot-and-mouth disease vaccine for pigs. Vaccines. (2024) 12:963. doi: 10.3390/vaccines12090963. PMID: 39339996 PMC11435775

[72] Choe S Park GN Song S Shin J Le VP Nguyen VG . Efficacy of needle-less intradermal vaccination against porcine epidemic diarrhea virus. Pathogens. (2021) 10:1115. doi: 10.3390/pathogens10091115. PMID: 34578148 PMC8471454

[73] Fourie KR Jeffery A Chand D Choudhary P Ng SH Liu H . Vaccination with a Lawsonia intracellularis subunit water in oil emulsion vaccine mitigated some disease parameters but failed to affect shedding. Vaccine. (2024) 42:126254. doi: 10.1016/j.vaccine.2024.126254. PMID: 39213981

[74] Jarosova R Ben Arous J Nechvatalova K Nedbalcova K Hlavova K Stepanova H . Effects of oil-based adjuvants on the immune response of pigs after dermal administration of antigen and evaluation of the immunization level after a subsequent Actinobacillus pleuropneumoniae challenge in pigs. Vet Microbiol. (2023) 276:109607. doi: 10.1016/j.vetmic.2022.109607. PMID: 36481482

[75] Qu S Dai H . Conjugated STING agonists. Mol Ther Nucleic Acids. (2025) 36:102530. doi: 10.1016/j.omtn.2025.102530. PMID: 40291379 PMC12032345

[76] Cong X Yuan Z Du Y Wu B Lu D Wu X . Crystal structures of porcine STINGCBD–CDN complexes reveal the mechanism of ligand recognition and discrimination of STING proteins. J Biol Chem. (2019) 294:11420–32. doi: 10.1074/jbc.RA119.007367. PMID: 31167783 PMC6663881

[77] Zhang X Shi H Wu J Zhang X Sun L Chen C . Cyclic GMP-AMP containing mixed phosphodiester linkages is an endogenous high-affinity ligand for STING. Mol Cell. (2013) 51:226–35. doi: 10.1016/j.molcel.2013.05.022. PMID: 23747010 PMC3808999

[78] Xia P Wang S Xiong Z Zhu X Ye B Du Y . The ER membrane adaptor ERAdP senses the bacterial second messenger c-di-AMP and initiates anti-bacterial immunity. Nat Immunol. (2018) 19:141–50. doi: 10.1038/s41590-017-0014-x. PMID: 29292386

[79] Pollock AJ Zaver SA Woodward JJ . A STING-based biosensor affords broad cyclic dinucleotide detection within single living eukaryotic cells. Nat Commun. (2020) 11:3533. doi: 10.1038/s41467-020-17228-y. PMID: 32669552 PMC7363834

[80] Hernandez-Franco JF Jan IM Elzey BD HogenEsch H . Intradermal vaccination with a phytoglycogen nanoparticle and STING agonist induces cytotoxic T lymphocyte-mediated antitumor immunity. NPJ Vaccines. (2024) 9:149. doi: 10.1038/s41541-024-00943-8. PMID: 39152131 PMC11329758

[81] Renu S Feliciano-Ruiz N Lu F Ghimire S Han Y Schrock J . A nanoparticle-poly(I:C) combination adjuvant enhances the breadth of the immune response to inactivated influenza virus vaccine in pigs. Vaccines. (2020) 8:229. doi: 10.3390/vaccines8020229. PMID: 32443416 PMC7349929

[82] Lorenzen E Follmann F Secher JO Goericke-Pesch S Hansen MS Zakariassen H . Intrauterine inoculation of minipigs with Chlamydia trachomatis during diestrus establishes a longer lasting infection compared to vaginal inoculation during estrus. Microbes Infect. (2017) 19:334–42. doi: 10.1016/j.micinf.2017.01.008. PMID: 28189786

