# Supplementary material for: A Chlamydia trachomatis CPAF-STING agonist conjugate vaccine administered intramuscularly and intradermally is immunogenic in the pig model
Source: Front Immunol. 2026 May 1;17:1816737. doi: 10.3389/fimmu.2026.1816737 (PMC13175861; doi:10.3389/fimmu.2026.1816737)
Supplement: Supplementary file 1 [file DataSheet1.pdf]

Supplementary Material

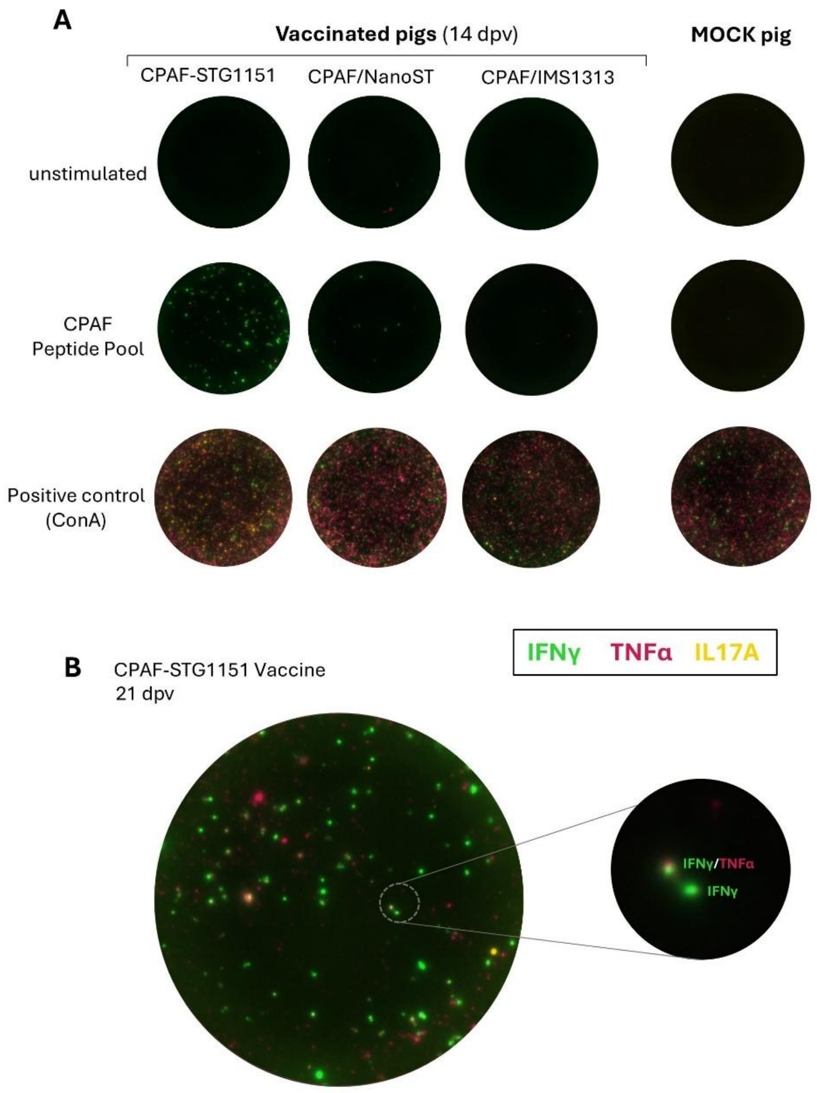

Supplementary Figure 1. Three-colour FluoroSpot analysis. Freshly isolated porcine PBMCs were cultured for two days and analyzed simultaneously for IFNγ, TNFα and IL-17A production. Panel **(A)** demonstrates the overlaid images under different restimulation conditions, namely unstimulated (media), CPAF peptide pool or positive control (ConA) for representative pigs from vaccinated or mock groups. IFNγ spots are shown in green, TNFα positive spots in pink and IL-17A positive spots in yellow. Each colored spot equals one SFU (spot forming unit) for the respective cytokine measured. Spots are analyzed for spot volume, spot intensity and location. Panel **(B)** shows an overlaid image from a representative animal vaccinated with the CPAF-STG1151 vaccine (21 dpv). Two spots, identified as a single and a dual cytokine-producing cell based on precise location parameters, are highlighted. dpv= days post (first) vaccination.

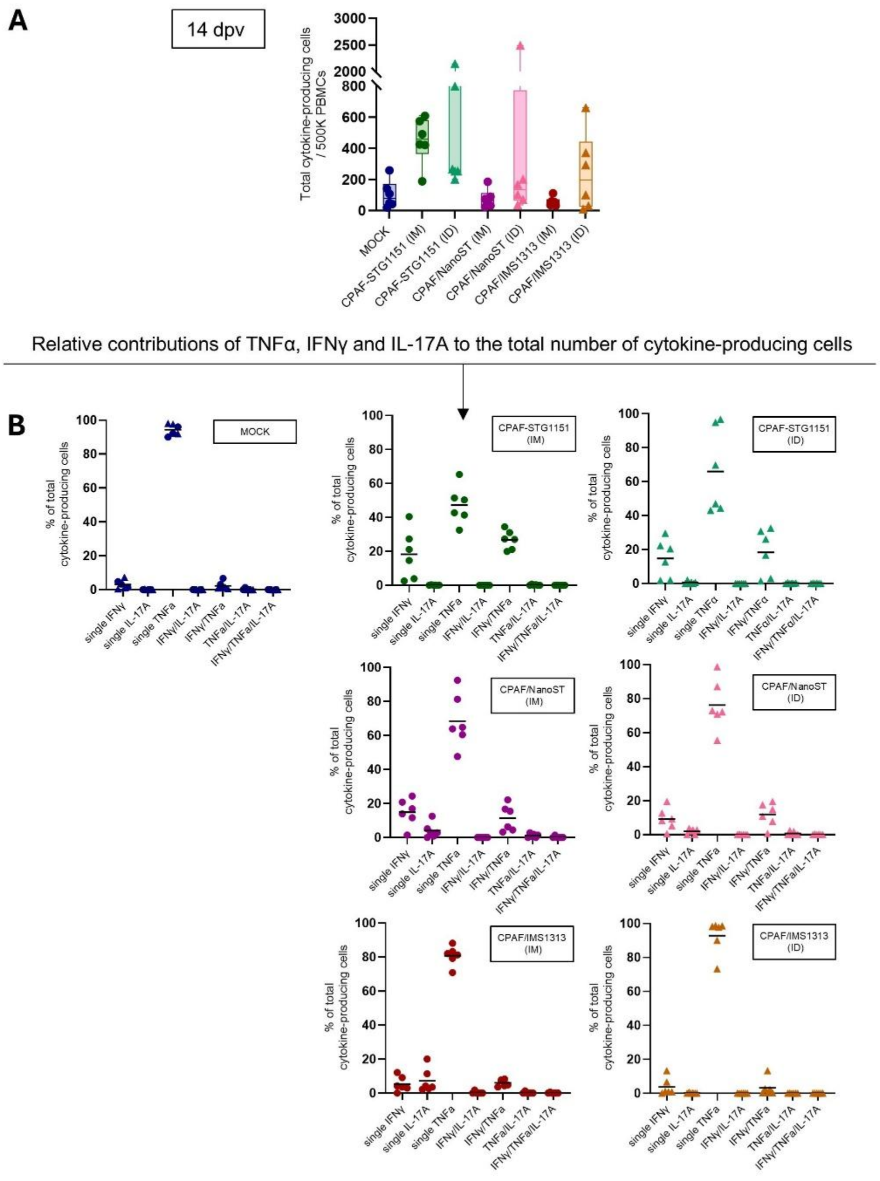

Supplemental Figure 2. The antigen-specific cytokine response after vaccination is characterized by IFNγ single and IFNγ/TNFα double producing cells. **(A)** shows the number of total cytokine producing cells (TNFα + IFNγ + IL-17A) 14 dpv measured by FluoroSpot assay after restimulation of freshly isolated PBMCs with CPAF peptide pool. No significant differences were observed between groups (one-way ANOVA and Tukey multiple comparisons test). **(B)** The frequency of all cytokine combinations within total cytokine producing cells in MOCK or vaccinated animals is shown for each group. In the MOCK group, three pigs received intramuscular (IM) PBS injections (circle), and three pigs received intradermal (ID) PBS injections (triangle). Each symbol represents an individual animal (n=6 per group). dpv= days post (first) vaccination.

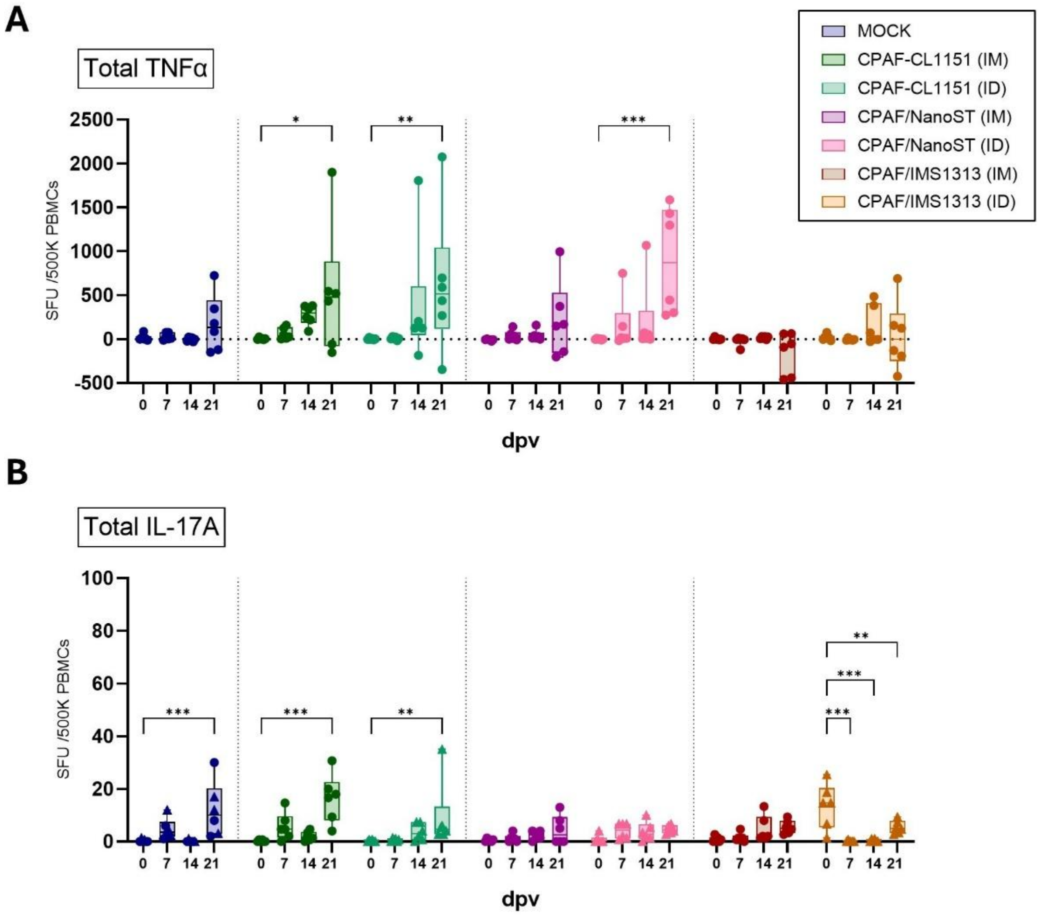

Supplementary Figure 3. TNFα and IL-17A production by PBMCs in response to *in vitro* CPAF restimulation. **(A)** Total TNFα production by PBMCs was measured by FluoroSpot assay after in vitro CPAF restimulation of freshly isolated PBMCs. Due to high TNFα counts in unstimulated control wells, the counts observed under CPAF peptide pool stimulation were background corrected by subtracting the SFU count in media (negative control, background) from the SFU count under CPAF stimulation. **(B)** shows the spot count for IL-17A positive cells. Each symbol represents an individual animal (n=6 per group). In the MOCK group, three pigs received intramuscular (IM) PBS injections (circle), and three pigs received intradermal (ID) PBS injections (triangle). Statistical analysis was performed via GraphPad using 2-way ANOVA and Tukey multiple comparisons test. The statistical analysis of within-group comparisons are shown * $p < 0.05$, ** $p < 0.01$, *** $p < 0.001$. dpv = days post (first) vaccination. SFU = spot forming units.

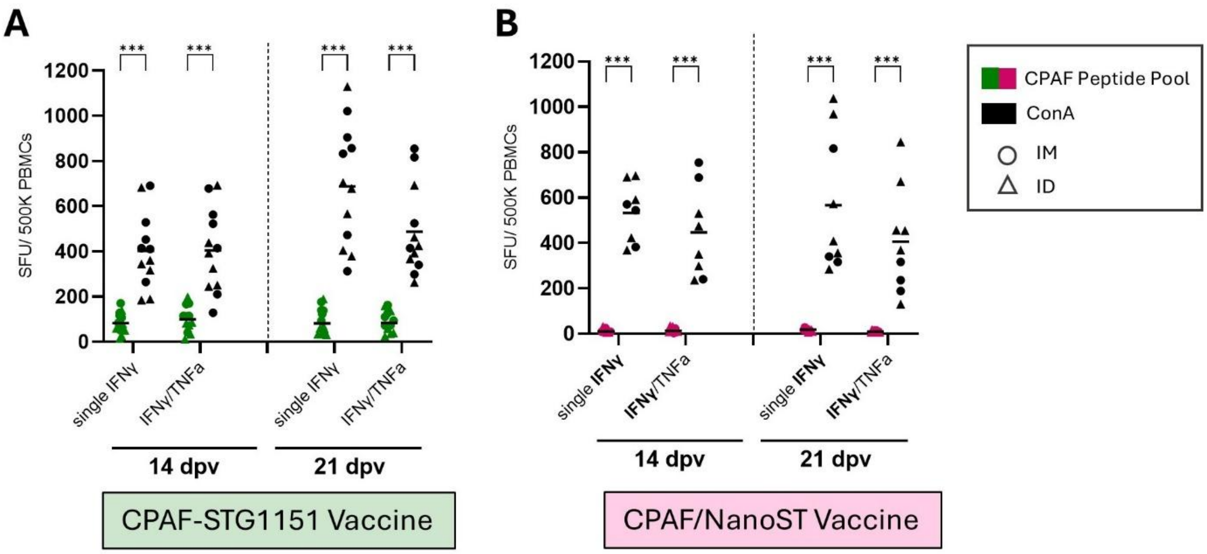

Supplementary Figure 4. IFNγ spot count of samples stimulated with CPAF peptide pool or ConA. The graphs show the IFNγ spot count in response to CPAF peptide pool stimulation and the positive control (ConA) at 14 and 21 dpv for pigs vaccinated with CPAF-STG1151 **(A)** or CPAF/NanoST **(B)**. IM and ID vaccinated pigs were combined for this analysis (n= 12 CPAF-STG1151 vaccine; n= 8 CPAF/NanoST vaccine at 14dpv and n= 9 at 21 dpv). Only responder samples were included in this analysis. The responder cut-off was set as the mean of the media control wells plus two standard deviations = 9.5 SFU / 500k PBMCs. Statistical analysis was performed via GraphPad using 2-way ANOVA and Tukey multiple comparisons test. *** $p < 0.001$. dpv = days post (first) vaccination. SFU = spot forming units.

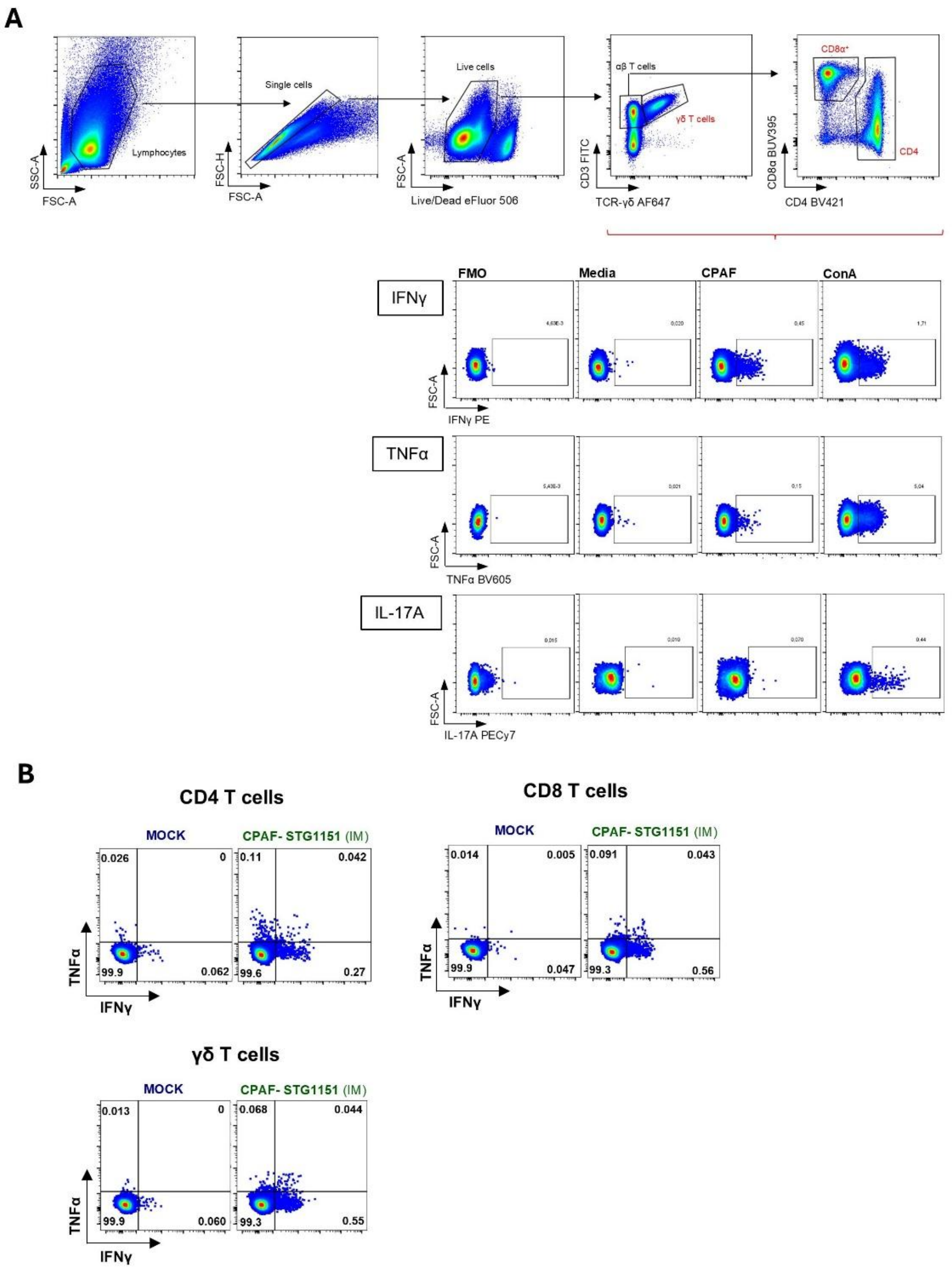

Supplementary Figure 5. Schematic representation of the gating strategy used to analyze cytokine production by T cell subsets. Lymphocytes were first identified, followed by singlet gating and exclusion of dead cells. Within the live cell population, γδ T cells were defined as TCR-$\gamma\delta^{+}$ and αβ T cells as $CD3^{+}TCR\text{-}\gamma\delta^{-}$. Alpha-beta T cells were further subdivided into $CD4^{+}$ and $CD8\alpha^{+}$ T cells. **(A)** Each T cell subset was then assessed for intracellular cytokine production (IFNγ , TNFα, IL-17A) under different stimulation conditions (media = negative control, CPAF = vaccine antigen, ConA = positive control). FMO = fluorescence minus one control. **(B)** shows representative flow plots for one MOCK and one CPAF-STG1151 vaccinated animal for the analysis of IFNγ and TNFα co-production within T cell subsets (CD4, CD8 and γδ T cells).

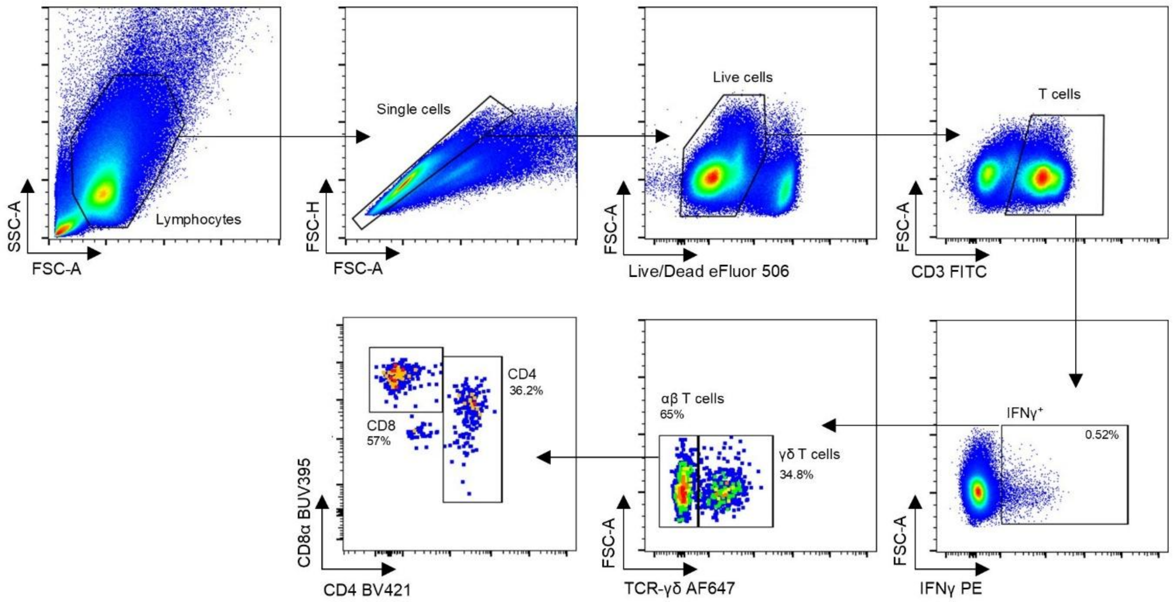

Supplementary Figure 6. Schematic representation of the gating strategy for the contribution of each T cell subset to the total IFNγ^{+} T cells. First, a gate was established around lymphocytes, followed by a gate for singlets before excluding dead cells. Within live cells, T cells were identified by the expression of CD3, followed by the gating on IFNγ positive cells. Based on the expression of the TCR-γδ, γδ T cells were separated from αβ T cells within the IFNγ^{+} T cells. Alpha-beta T cells were then further subdivided into CD4^{+} and CD8^{+} T cells.

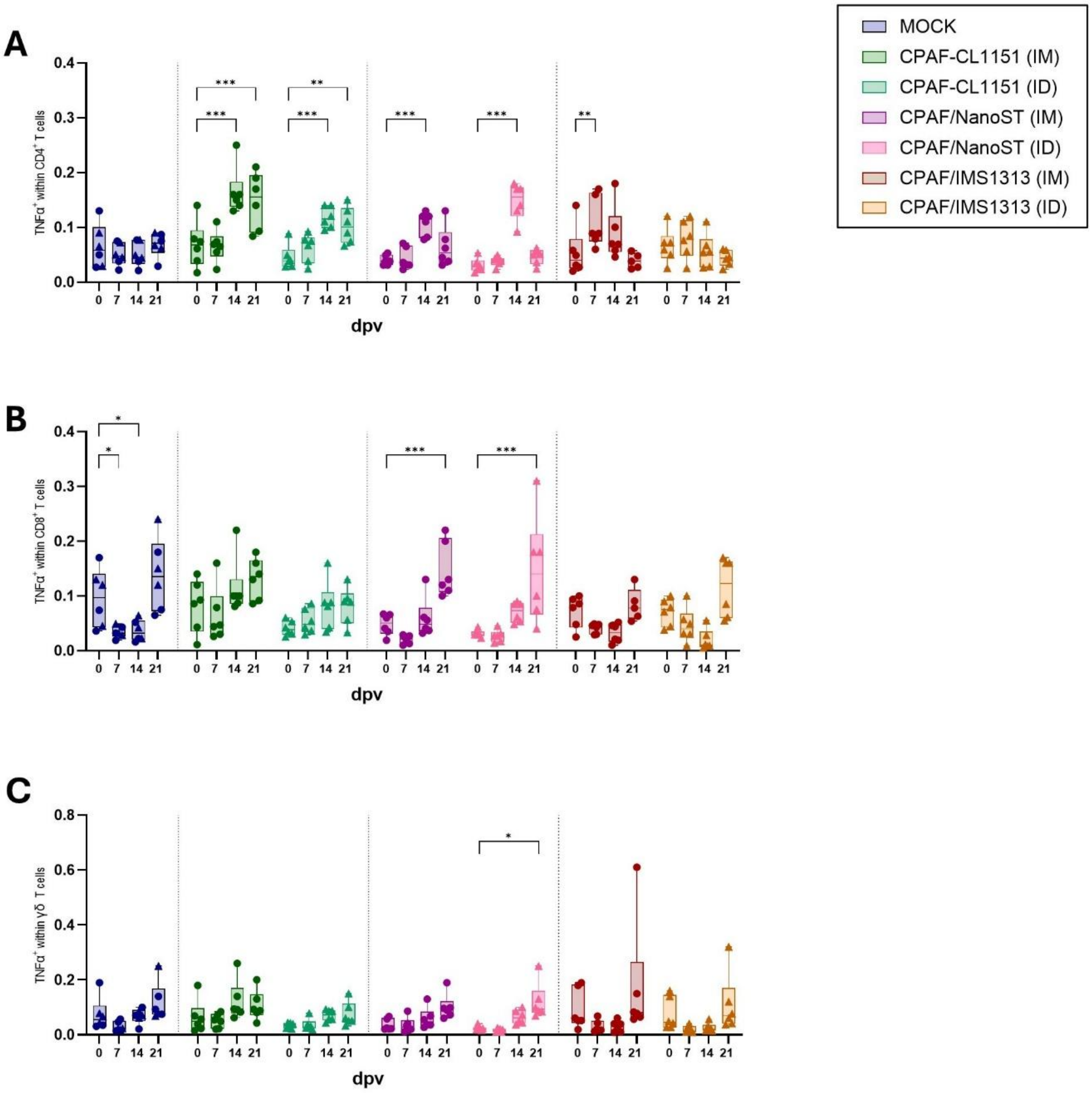

Supplementary Figure 7. TNF-α production within T cell subsets in response to in vitro CPAF restimulation. Freshly isolated PBMCs were restimulated in vitro with CPAF peptide pool. During data analysis, dead cells and doublets were excluded, as shown in **Supplementary Figure 5**. After identification of T cell subsets, their TNF-α production was analyzed. The scatter diagrams show the percentage of TNF-α positive cells within CD4 (A), CD8 (B) and γδ T cells (C) at different time points and for all groups. Each symbol represents data from one individual pig (n= 6 per group). In the MOCK group, three pigs received intramuscular (IM) PBS injections (circle), and three pigs received intradermal (ID) PBS injections (triangle). Statistical analysis was performed via GraphPad using 2-way ANOVA and Tukey multiple comparisons test. The statistical analysis of within-group comparisons are shown. * $p < 0.05$, ** $p < 0.01$, *** $p < 0.001$. dpv= days post (first) vaccination.

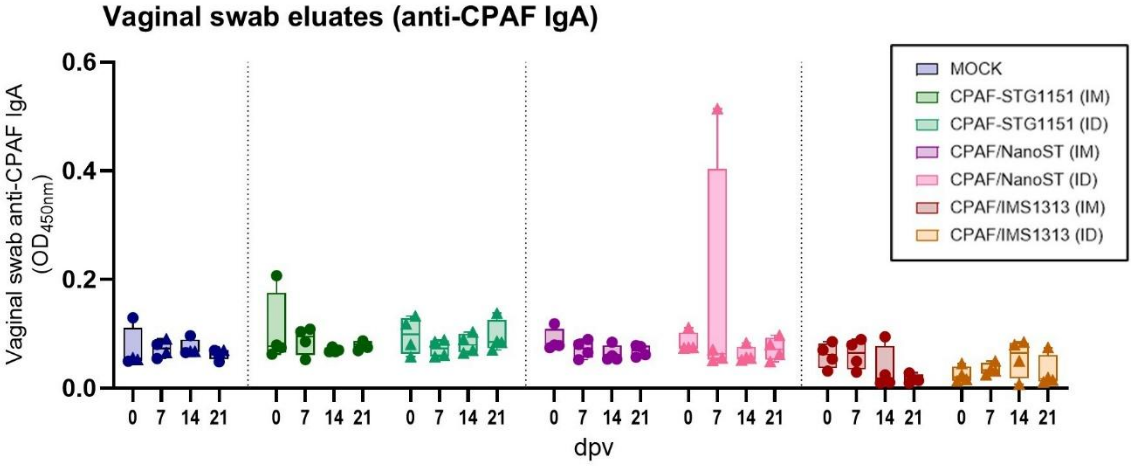

Supplementary Figure 8. Evaluation of vaginal swab eluates for anti-CPAF IgA. Vaginal swab eluates from CPAF-STG1151, CPAF/NanoST, and CPAF/IMS1313 vaccinated pigs (female) were analyzed by ELISA for CPAF-specific IgA responses. Each symbol represents data from one individual pig (n=4 per group). In the MOCK group, two pigs received intramuscular (IM) PBS injections (circle), and two pigs received intradermal (ID) PBS injections (triangle). Statistical analysis was performed via GraphPad using 2-way ANOVA and Tukey multiple comparisons test. dpv= days post (first) vaccination.

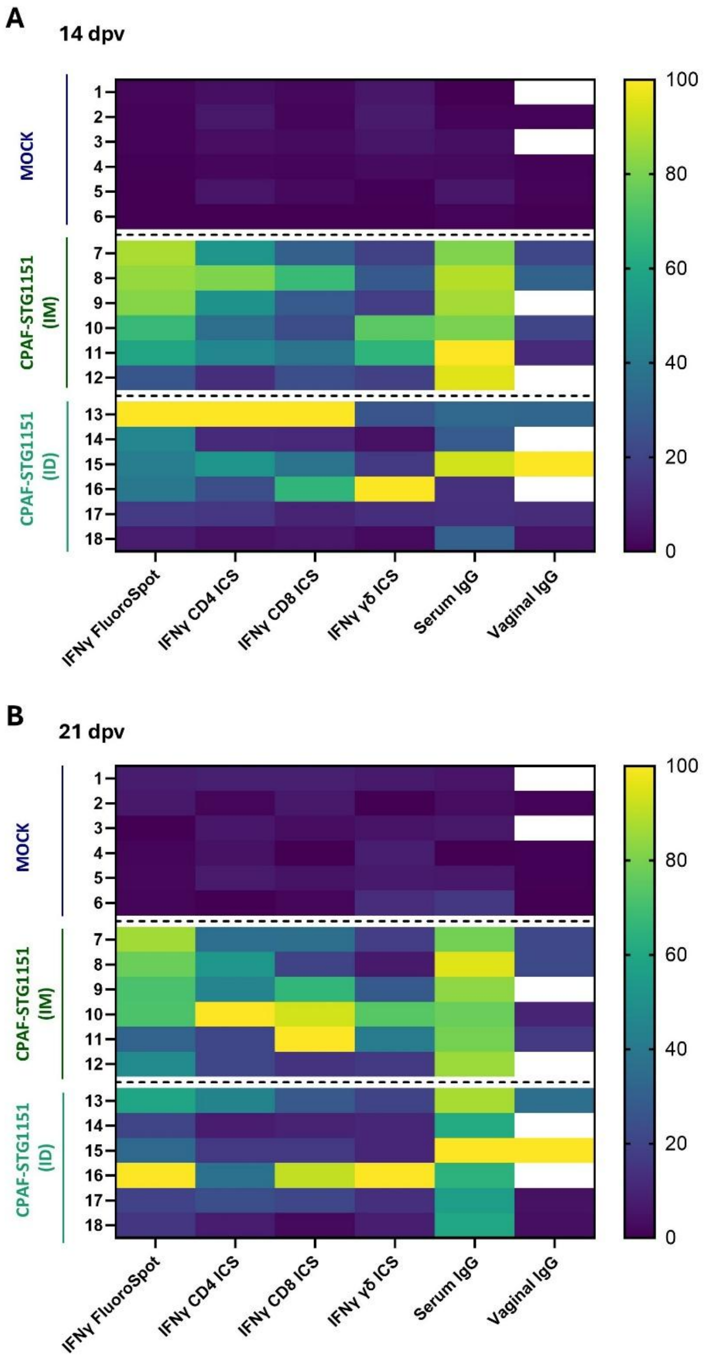

Supplementary Figure 9. Heatmap illustrating individual responses across all readouts. Data from the IFNγ FluoroSpot assay (SFU/ 500k following CPAF restimulation), intracellular cytokine staining (frequency of IFNγ+ cells within CD4, CD8 and γδ T cells after CPAF restimulation) and anti-CPAF IgG levels in serum and vaginal swab eluates (OD values) are shown for individual pigs. Animals are grouped as follows: MOCK (pigs 1-6), CPAF-STG1151 IM (pigs 7-12), CPAF-STG1151 ID (pigs 13-18). Row labels show assigned animal number. Responses were normalized within each assay to the highest observed value, which was set to 100%, with all other responses expressed relative to this maximum. **(A)** shows the individual responses across readouts at 14 dpv. **(B)** shows the individual responses across readouts at 21 dpv. Responses were ranked based on the IFNγ FluoroSpot assay at 14 dpv **(A)** and the same animal order was maintained in **(B).**
